# Lattice oxygen insertion mechanism in CeO_2_-catalyzed reactions in water: nitrile hydration reaction†

**DOI:** 10.1039/d4sc06294a

**Published:** 2024-12-02

**Authors:** Takaaki Endo, Tatsushi Ikeda, Koki Muraoka, Yusuke Kita, Masazumi Tamura, Akira Nakayama

**Affiliations:** a Department of Chemical System Engineering, The University of Tokyo Tokyo 113-8656 Japan nakayama@chemsys.t.u-tokyo.ac.jp; b Department of Chemistry and Bioengineering, School of Engineering, Osaka Metropolitan University 3-3-138, Sugimoto, Sumiyoshi-ku Osaka 558-8585 Japan mtamura@omu.ac.jp

## Abstract

Cerium oxide (CeO_2_) exhibits prominent catalytic activity in various organic reactions owing to its unique acid–base and redox properties. One of the most interesting applications of pure CeO_2_-catalyzed organic reactions is the hydration of nitriles in water. The experimental results showed that the hydration of 2-cyanopyridine to picolinamide in water using CeO_2_ catalysts proceeds readily at low temperatures (30–100 °C) in high yields and that this reaction occurs exclusively on CeO_2_ among various metal-oxide catalysts. To elucidate the unique catalytic activity of CeO_2_, the reaction mechanism is dissected using the density functional theory-based molecular dynamics (DFT-MD) simulations. Based on the free energy analysis, it is demonstrated that the reaction proceeds with the involvement of the surface lattice oxygen, where the lattice oxygen atom is inserted into picolinamide. The involvement of the surface lattice oxygen is notably uncommon given the low temperatures of the reaction, and this computational prediction is verified by the two experiments using H_2_^18^O solvent and ^18^O-exchanged CeO_2_ catalyst, where the introduction of surface lattice oxygen into picolinamide is confirmed. The inherent flexibility of the surface lattice oxygen and the unique acid–base properties of CeO_2_, which can favorably bind and activate both nitrile and water molecules, are key factors in the high reactivity for various organic reactions, which characterizes the outstanding catalytic activity of CeO_2_.

## Abstract 2

The reaction mechanism of hydration of 2-cyanopyridine to picolinamide in water over CeO_2_ catalysts is dissected using the DFT-MD simulations. It is demonstrated that the lattice oxygen atom is inserted into picolinamide.

## Graphical abstract

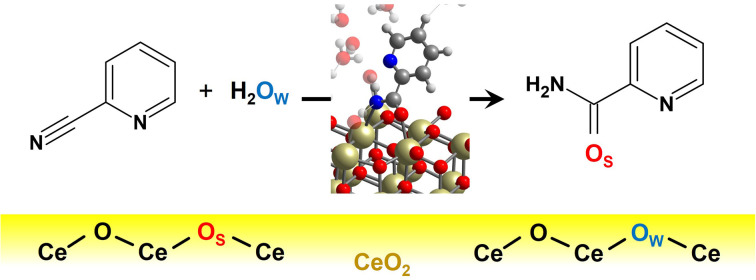

## 1. Introduction

Among various metal-oxides, cerium oxide (CeO_2_) has received considerable attention because of its immense scientific and technological importance in fields such as catalysis, glass polishing, biomedical technology, and sensors. In particular, the number of catalytic applications of CeO_2_ is continuously increasing, and CeO_2_ is extensively used in crude oil refining, solid oxide fuel cells (SOFC), vehicle emission control, and so on. The wide applicability of CeO_2_ is attributed to its unique redox and acid–base properties, oxygen storage capacity, and high thermal stability and durability (see ref. 1–6 for the recent reviews). In the catalytic applications of CeO_2_, its high reducibility and basicity promote the activation and dispersion of metal nanoparticles *via* strong metal-oxide electronic interactions,^7–9^ and CeO_2_-supported metal nanoparticles have a wide range of catalytic applications.^10–14^ Even without supported metal nanoparticles, pure CeO_2_ is also quite effective for various organic reactions (see the reviews in ref. 15–17), which characterizes CeO_2_ as a highly unique metal-oxide.

**Fig. 1:**
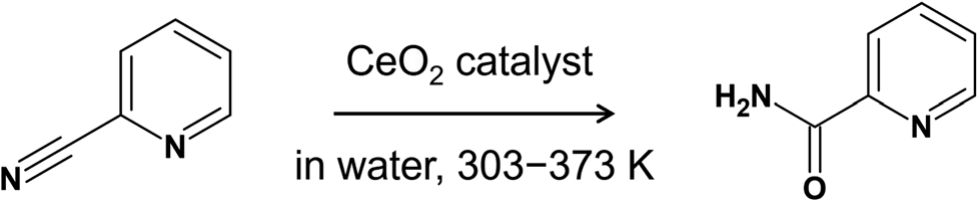
Hydration of 2-cyanopyridine to picolinamide over CeO_2_ catalyst.

In one of the most interesting applications of pure CeO_2_-catalyzed organic reactions, Tamura *et al.* reported that the hydration of 2-cyanopyridine to picolinamide over CeO_2_ proceeds very smoothly at low temperatures (30–100 °C) with high yields (Fig. 1).^18,19^ It was also found that this reaction proceeds exclusively on CeO_2_ among various metal-oxide catalysts and that the activity is two orders of magnitude higher than that of other metal-oxides. Based on this finding, 2-cyanopyridine was later used as a dehydration agent for the direct conversion of CO_2_ and methanol to dimethyl carbonate on CeO_2_ catalysts, where 94% yield and 99% selectivity were reported.^20^ Also, it was further utilized in CO_2_ conversion with alcohols and amines into carbonates, ureas, carbamates over CeO_2_.^21,22^ In our previous works on the adsorption of 2-cyanopyridine over CeO_2_(111),^23–26^ we found a unique adsorption structure of 2-cyanopyridine, involving a covalent interaction between the C atom in the CN group of 2-cyanopyridine and the surface lattice O atom along with the acid–base interaction between the N atoms in the pyridine ring and the surface Ce atom. This unique adsorption structure creates a strong base site at the position of the N atom in the CN group, and it has been demonstrated that base-catalyzed reactions proceed effectively and that the p*K*_a_ (CH_3_CN) is estimated to be ∼21.^23^ In another related study, we examined the adsorption structure of picolinic acid over CeO_2_(111) and found a similar adsorption structure that involves a covalent bond between the C atom in the carboxylic group and the surface O atom.^27^ Interestingly, when we further performed the DFT-based molecular dynamics simulations (DFT-MD) to gain insight into the adsorption process in water, the spontaneous dissociation of a hydroxide ion from the carboxylic group was observed, which implies the rich chemistry at the interface of water and CeO_2_ surface.

These observations motivated us to investigate the reaction mechanism of the hydration of 2-cyanopyridine over CeO_2_ by employing DFT-MD simulations with explicit solvent water molecules. The DFT-MD simulations are now widely used as an invaluable tool to obtain a bottom-up picture of the liquid/metal-oxide interface and thus provide useful information on the microscopic properties and dynamical behavior of molecules at the interfaces. In the previous studies by the DFT-MD simulations at the water/CeO_2_ interface, high activity at the interface involving proton hopping was reported.^28–31^ In this study, the reaction mechanisms of nitrile hydration on the CeO_2_(111) surface are examined by computing the free energy landscape, and we decipher the role of CeO_2_ in these unique catalytic reactions. Based on the free energy analysis, we find that the reaction proceeds with the involvement of the surface lattice oxygen, and this computational prediction is further verified by the two experiments using H_2_^18^O solvent and ^18^O-exchanged CeO_2_ catalyst.

## 2. Computational details

### 2.1. DFT calculations

All DFT calculations were performed under the periodic-boundary condition with the mixed Gaussian and plane-waves (GPW) approach implemented in the CP2K program package.^32^ The short-range variants of the double-ζ valence plus polarization (DZVP) basis sets of the MOLOPT type^33^ were employed for H, C, N, and O atoms to represent the valence electrons, and the norm-conserving Goedecker–Teter–Hutter pseudopotentials^34,35^ were used to describe the interactions between the valence and core electrons. For Ce atoms, we employed the basis sets and pseudopotential generated by Wang and coworkers.^36^ The energy cutoff of 400 Ry was taken for the auxiliary plane wave expansion of the density. The generalized-gradient approximation by the Perdew–Burke–Ernzerhof (PBE) functional models was employed as the exchange and correlation potential, and the DFT+U approach was taken in order to correctly represent the nature of 4f orbitals of Ce atoms, where the occupancies of 4f orbitals were calculated based on the Mulliken population analysis.^37,38^ The *U* value was set to 7.0 eV following the previous works.^36^ The Brillouin zone integration was performed with a reciprocal space mesh consisting of only the Γ-point in the GPW approach. The convergence criteria for the energy in the SCF calculation were set to 1 × 10^−6^ hartree.

Cerium oxide exhibits fluorite structure in the low-temperature regime. It has been determined theoretically^39^ and experimentally^40^ that the CeO_2_(111) surface is the most stable low-index surface among single crystal terminations of CeO_2_, and therefore it represents a large portion of the polycrystalline CeO_2_ surface.^39,41^ CeO_2_-catalyzed organic reactions are often reported to be influenced by the exposed facets.^42^ In our catalyst system in the nitrile hydration, the activity per surface area does not change significantly even after high-temperature calcination,^18^ suggesting that the main active facet is the (111) facet. Furthermore, a recent study has demonstrated that the (111) facet exhibits high activity for the hydration of 2-cyanopyridine.^43^ Therefore, the surface of the CeO_2_(111) facet was employed in this study. The CeO_2_(111) surface was modeled as a periodic *p*(3 × 3) hexagonal slab of 27 CeO_2_ units with three O–Ce–O trilayers. The dimensions of a simulation cell were set to *a* = *b* = 11.56 Å, *c* = 30.0 Å and *α* = *β* = 90°, *γ* = 60°, and this slab was separated by ∼16 Å of vacuum space in the direction perpendicular to the surface. The lattice parameters of the CeO_2_(111) slab were determined by the cell optimization of the bulk CeO_2_, and the optimized lattice constant of 5.450 Å was in good agreement with the experimental value of 5.411 Å,^44,45^ where the deviation from the experimental value was less than 1%. For geometry optimization, the forces on all atoms are minimized to less than 0.02 eV Å^−1^ (4.5 × 10^−4^ hartree bohr^−1^). The bottom O–Ce–O tri-layers were fixed at the bulk positions during geometry optimization and DFT-MD simulations. In calculating the energies of gas-phase molecules, the simulation cell of *a* = *b* = *c* = 15.0 Å in the cubic box is used.

The adsorption energy of a molecule *E*_A_ to the CeO_2_(111) surface is calculated according to

1 *E*_A_ = *E*_mol+surf_ − *E*_mol_ − *E*_surf_

where *E*_mol+surf_ is the total electronic energy of the surface-molecular system while *E*_surf_ and *E*_mol_ are the energy of the pristine CeO_2_ slab model and an isolated molecule, respectively. In this definition, the more negative value of adsorption energy indicates a stronger binding to the surface.

### 2.2. Molecular dynamics simulations with umbrella sampling

For the investigation of chemical reactions that occur on a much longer timescale than that allowed in DFT-MD simulations (so-called rare events), free energy calculations are generally carried out. Computing the free energy landscape and finding the lowest free energy pathway on this landscape provide valuable information on the mechanism and kinetics of the reaction.^46^ The free energy landscape is generally represented by a few selected coarse-grained coordinates or collective variables (CVs), and several advanced sampling techniques have been proposed to explore the free energy landscape along these CVs: these include metadynamics,^47,48^ umbrella sampling,^49^ and several others.^50–52^

In this work, we employ the umbrella sampling techniques for constructing free energy landscape. Two types of CVs are used in this work. The first one is the distance between atoms *i* and *j*, and it is given as

2 *d*_*ij*_  =  |**R**_*i*_  −  **R**_*j*_|

The second one is a coordination number, and the coordination number between two atom types (X and Y) is defined as3
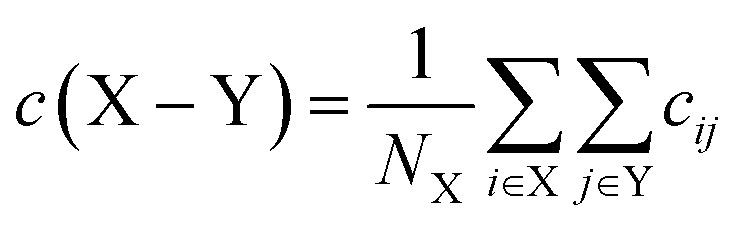
where *N*_X_ is the number of atoms with atom type X, and *c*_*ij*_ is a so-called switching function and represented as4
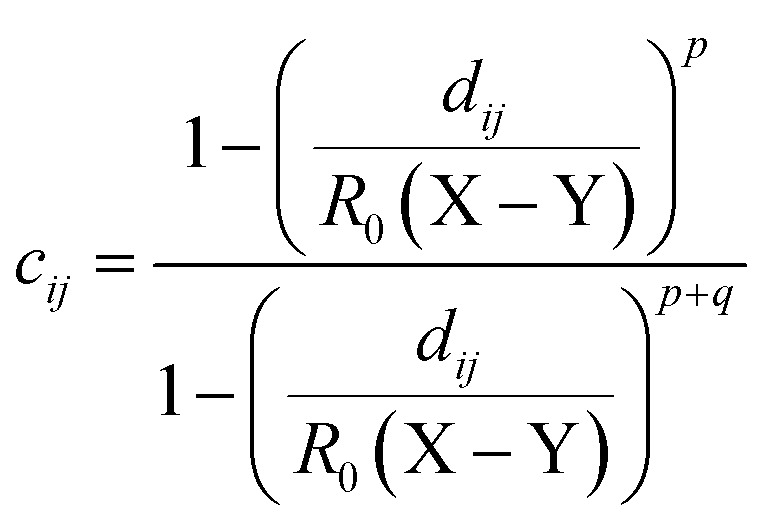
where *d*_*ij*_ is the distance between atoms *i* and *j* belonging to the atom types X and Y, respectively, and *p* and *q* are parameters to define the shape of the switching function. *R*_0_(X − Y) is a cutoff bond distance between atom types X and Y. More details are given in the Results and discussions section.

In the following simulations, 32 water molecules were placed over the CeO_2_(111) surface in addition to 2-cyanopyridine. The initial configurations of water molecules were generated by the GROMACS package.^53^ The DFT-MD simulations were performed at a system temperature of *T* = 360 K with the umbrella potential, where quadratic potentials centered at predefined points were applied. The temperature was controlled by the Nosé–Hoover thermostat. The mass of hydrogen was replaced with that of deuterium, allowing for a larger time step of 1.0 fs. For each umbrella window, 10 ps simulations were performed for equilibrium and subsequent 10 ps simulations were used for analysis, which is sufficient to ensure the dissociative equilibrium of water molecules.^28,29,31^ Around the transition state region, additional umbrella sampling was performed using the configuration of adjacent window that is closer to the transition state region as an initial configuration, and the convergence of probability distributions was confirmed. The biased probability distributions from different umbrella windows were reweighted and patched by the weighted histogram analysis method (WHAM)^54^ using the implementation by Grossfield.^55^ The PLUMED package^56^ interfaced with CP2K was used for the umbrella sampling. Free energy barrier heights were estimated using multidimensional lowest energy (MULE) algorithm code implemented by Fu *et al.*^57^

## 3. Results and discussions

In this section, the reaction mechanism of hydration of 2-cyanopyridine over CeO_2_ is investigated. Experimentally, the following reaction mechanism has been proposed:^18^ (1) dissociation of water molecules on CeO_2_ giving rise to H^*δ*+^ and OH^*δ*−^ pair species, (2) formation of adsorption complex between 2-cyanopyridine and CeO_2_, (3) addition of OH^*δ*−^ to the C atom in the CN group *via* an amide anion intermediate, and (4) desorption of the amide product (picolinamide) from the CeO_2_ surface accompanying regeneration of the adsorption site. Since no kinetic isotopic effect was observed (*k*_H_/*k*_D_ = 0.95), meaning that the water dissociation is fast on CeO_2_, the third step is considered to be the rate-determining step. As already reported in the literature,^28–31^ water molecules are activated on CeO_2_ and about 20–33% of the surface oxygen sites are hydroxylated (degree of hydroxylation). Since adsorption energy of a water molecule in the molecular and dissociative states are quite similar, proton hopping is quite active at the water/CeO_2_(111) interface.^28–31^ Therefore, the presence of surface hydroxy groups does not impede the approach and adsorption of 2-cyanopyridine on the surface. Consequently, we consider steps (2) and (3) in the following analysis.

### 3.1. Adsorption process of 2-cyanopyridine over CeO_2_

**Fig. 2:**
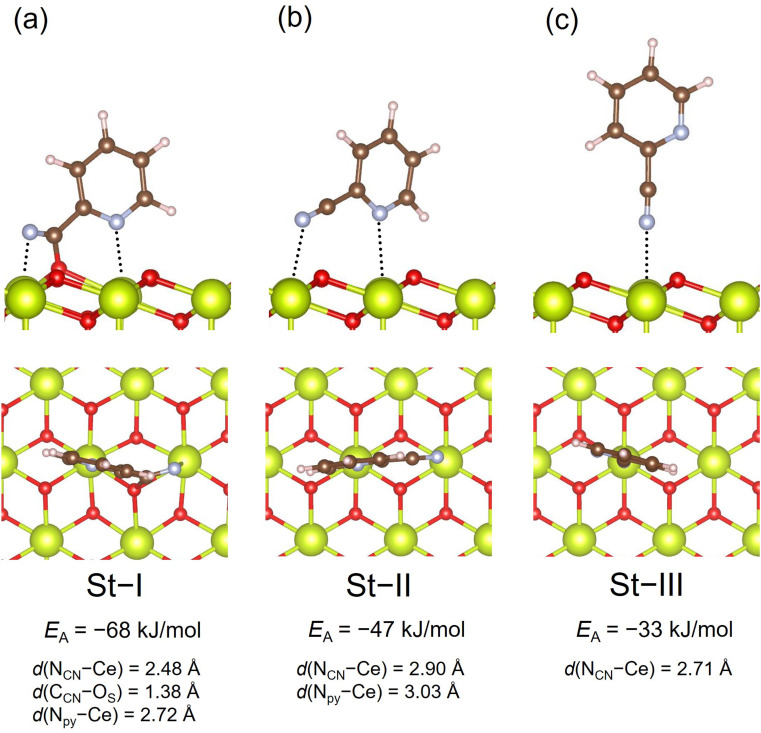
Adsorption structures of 2-cyanopyridine on CeO_2_(111) (a) St-I, (b) St-II, and (c) St-III.

The adsorption structures of 2-cyanopyridine over CeO_2_(111) have already been reported,^23^ and there are three types of adsorption states, which are shown in Fig. 2. The strongest adsorption involves the covalent bond between the C atom in the CN group (C_CN_) and the surface lattice O atom (O_S_), along with the acid–base interaction between the N atom in the pyridine ring (N_py_) and the surface Ce atom (denoted by St-I). The adsorption energy of St-I was calculated to be −68 kJ mol^−1^. It has been known that this adsorption structure forms a strong base site in the CN position of 2-cyanopyridine.^23^ Another adsorption structure (denoted by St-II) involves molecular adsorption where 2-cyanopyridine interacts with the surface Ce atoms through the two N atoms in the pyridine ring and the CN group with a distance of ∼3.0 Å, where the adsorption energy was −47 kJ mol^−1^. St-III involves the acid–base interaction only between the N atom in the CN group and the surface Ce atom with the adsorption energy of −33 kJ mol^−1^.

**Fig. 3:**
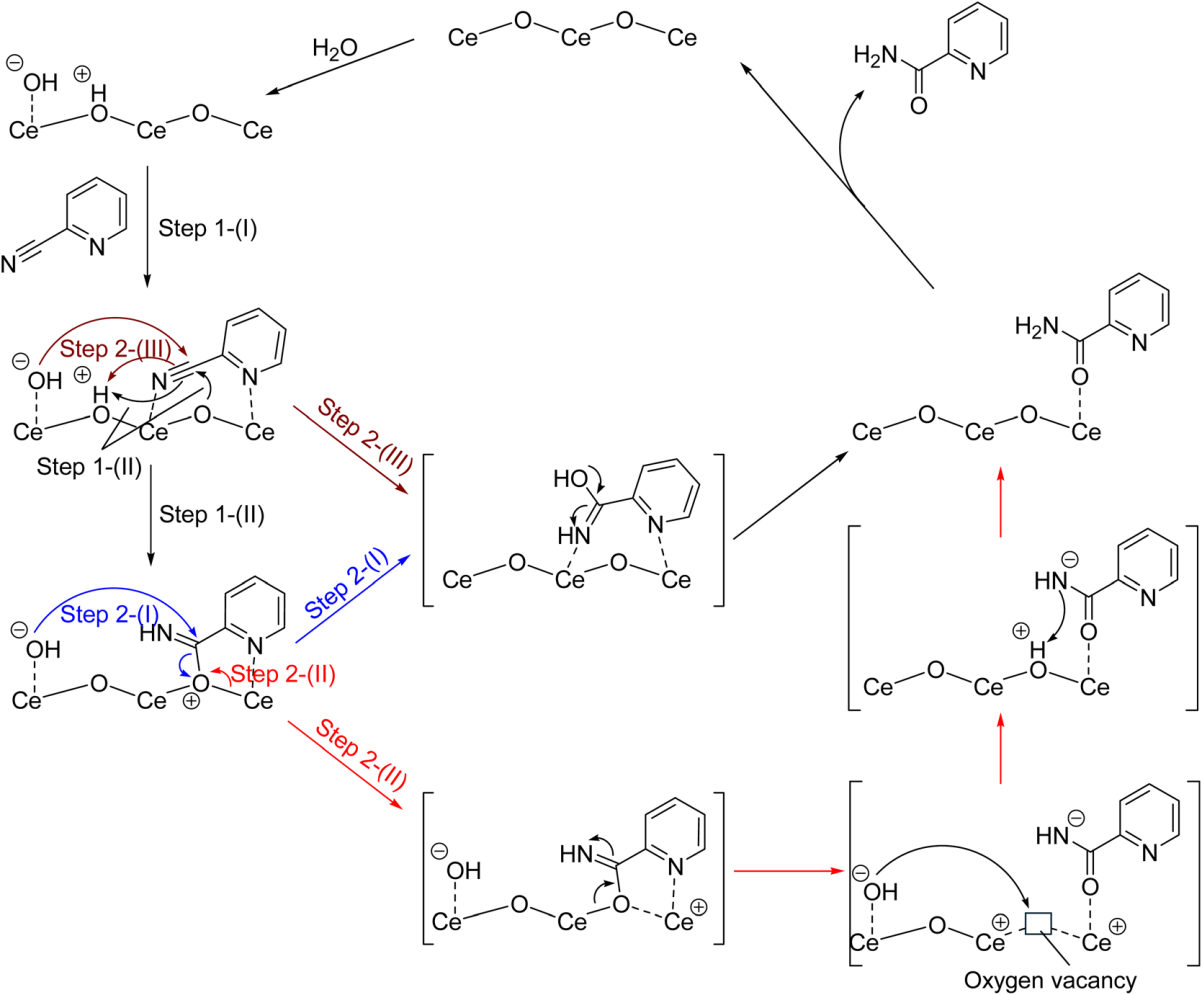
Schematics of reaction mechanisms.

The adsorption process of 2-cyanopyridine from the molecular adsorption structure, which corresponds to St-II, to the structure with the C_CN_–O_S_ covalent bond (corresponding to St-I) is then investigated in water environment by DFT-MD simulations with umbrella sampling (hereinafter referred to as Step 1-(II)). When we performed the standard DFT-MD simulation starting from St-I with surrounding water molecules, a proton was spontaneously transferred to the N atom in the CN group (N_CN_) from an adjacent water molecule due to the strong basicity of the N_CN_ atom formed by this unique adsorption structure of St-I.^23^ The resulting hydroxide ion then accepts a proton from a nearby water molecule, and subsequently, a hydroxide ion is transferred to the Ce atom after a couple of proton hopping events *via* the Grotthuss-like diffusion mechanism. This implies that during the adsorption process from St-II to St-I in water environment, a proton is transferred to the N atom in the CN group. The schematic representation is given in Fig. 3. For the sake of simplicity, a proton transferred to the N atom is depicted as originating from surface hydroxy group, although in DFT-MD simulations it originates from a nearby water molecule, as mentioned above. To obtain the free energy profile for this process, two CVs were introduced. The first CV (CV1) was set as the distance between C_CN_ and one of the surface O_S_ atoms, *d*(C_CN_–O_S_), and the second CV (CV2) was chosen as *c*(N_CN_–H_W_), where H_W_ is hydrogen atoms of water molecules, to promote proton transfer events between N_CN_ and the surrounding water molecules. For CV2, the cutoff bond distance was set to *R*_0_(N_CN_–H_W_) = 1.2 Å, and *p* = 6 and *q* = 6 were used to define the shape of the switching function. The umbrella potentials were placed between 1.3 Å and 3.1 Å for CV1, and those of CV2 were chosen between 0.1 and 0.9. The force constants of CV1 and CV2 were set to 20.7 eV Å^−2^ and 31.1 eV, respectively. For CV2, a larger force constant of 41.5 eV was used near the transition state region.

**Fig. 4:**
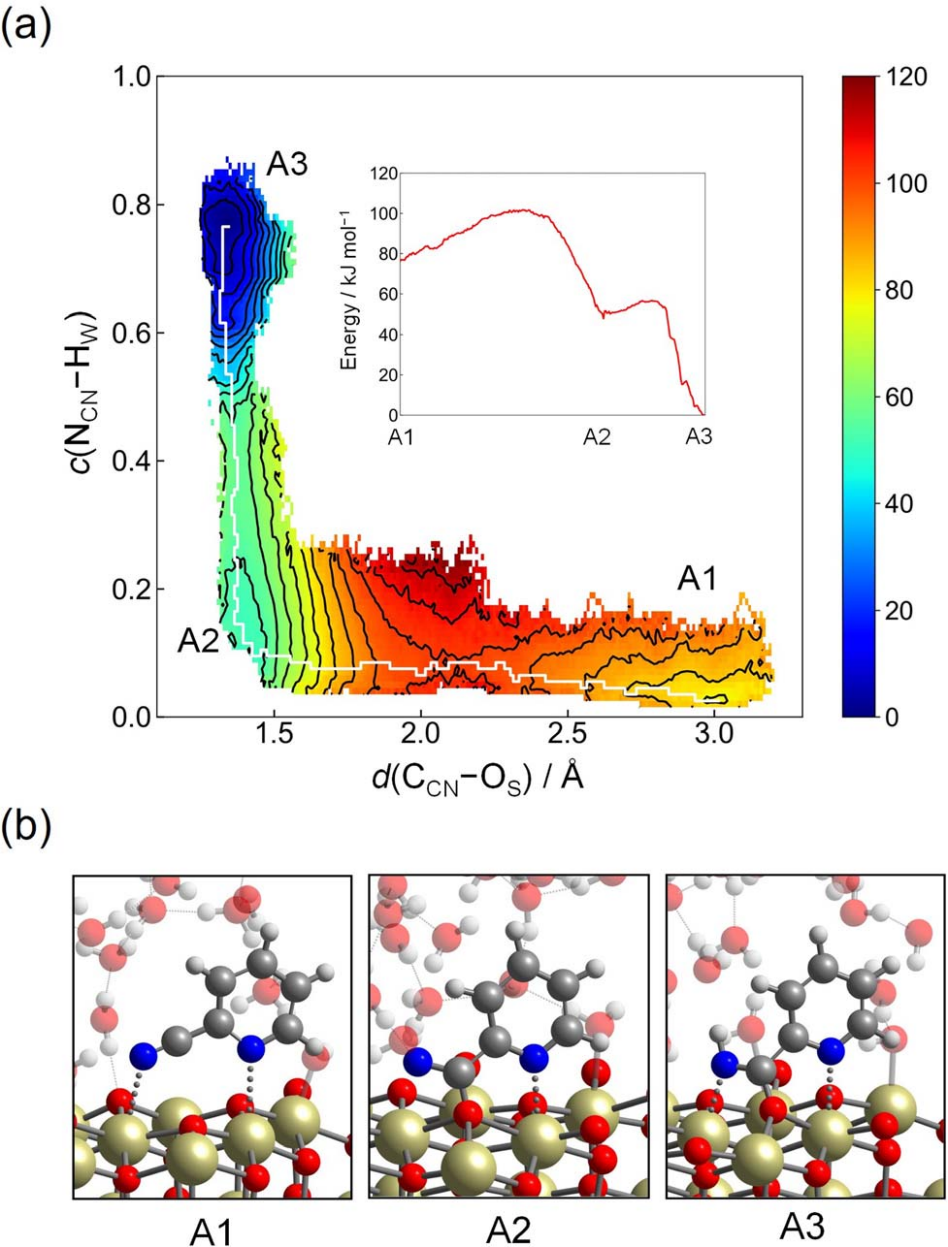
(a) Contour plot of the free energy surface (given in kJ mol^−1^) for adsorption process of 2-cyanopyridine from the molecular adsorption state (Step 1-(II)). The minimum free energy pathway, which is determined by the MULE algorithm, is shown in white. The inset shows the free energy profile along the minimum free energy pathway. (b) Snapshots of representative configuration near A1, A2, and A3.

The free energy surface was constructed using CV1 and CV2, and the two-dimensional contour plot of the free energy surface along CV1 = *d*(C_CN_–O_S_) and CV2 = *c*(N_CN_–H_W_) is shown in Fig. 4(a). Starting from the region corresponding to the molecular adsorption state at *d*(C_CN_–O_S_) ≈ 3.0 Å (A1, the snapshot is given in Fig. 4(b)), the molecule approaches the surface by surmounting a small free energy barrier of 25 kJ mol^−1^ at *d*(C_CN_–O_S_) ≈ 2.1 Å. After passing through this barrier, the free energy surface reveals a metastable state at *d*(C_CN_–O_S_) ≈ 1.4 Å and *c*(N_CN_–H_W_) ≈ 0.1 (A2), representing the adsorption state with a covalent C_CN_–O_S_ bond. This structure corresponds to St-II in Fig. 2. Then, there is a small barrier for a proton transfer from a neighboring water molecule, which is not an adsorbed one, to the N_CN_ atom, leading to the stable protonated adsorption state (A3) with *d*(C_CN_–O_S_) ≈ 1.4 Å and *c*(N_CN_–H_W_) ≈ 0.75. The free energy difference between the molecular adsorption state (A1) and protonated adsorption state (A3) is 76 kJ mol^−1^, which indicates a strong adsorption of protonated 2-cyanopyridine. Considering the difference in adsorption energy between St-I and St-II given in Fig. 2 (21 kJ mol^−1^), the large stabilization of A3 is mainly due to the protonation of 2-cyanopyridine. The barrier height for the adsorption process is close to the values obtained in a similar simulation for the adsorption of picolinic acid to the CeO_2_(111) surface (∼30 kJ mol^−1^),^27^ which also involves the C_CN_–O_S_ covalent bond formation.

Here we also examine the adsorption process of 4-cyanopyridine where it is experimentally shown that the reaction rate of hydration of 4-cyanopyridine is 10^−7^-order of magnitude smaller than that of 2-cyanopyridine. The free energy surface of the adsorption process was constructed in the same way as above (the details are provided in ESI†), and it was found that the free energy barrier leading to the product state was 67 kJ mol^−1^, which is much higher than that of 2-cyanopyridine. This is mainly due to the steric hindrance between the pyridine ring and the surface, and the unfavorable adsorption of 4-cyanopyridine is related to the very low activity of the hydration reaction.

### 3.2. Hydration of 2-cyanopyridine on CeO_2_

The hydration reaction is next investigated from the above adsorption state as a protonated 2-cyanopyridine species (A3). Two reaction mechanisms are considered. The first mechanism involves the nucleophilic attack of hydroxide ion that is formed on the nearby Ce atom to the C_CN_ atom (Step 2-(I)), while the second one involves the surface O_S_ atom where the surface O_S_ atom is released from the surface and introduced into picolinamide (Step 2-(II)). The second mechanism has not been considered because the surface O_S_ atoms have not been thought to be involved in the reaction at low temperatures (below room temperature). The two reaction mechanisms are schematically shown in Fig. 3.

In the first mechanism (Step 2-(I)), we consider the reaction mechanism where a hydroxide ion that is formed on the Ce atom nucleophilically attacks the C_CN_ atom. Here, the following two CVs were introduced for the free energy analysis: CV1 = *d*(C_CN_–O_W_) and CV2 = *c*(O_W_–H_W_). CV1 corresponds to the distance between the C_CN_ atom and one of the O atoms deriving from water molecules, which promotes the nucleophilic attack of the hydroxide ion. CV2 was introduced to accelerate the deprotonation of the attacking water on the surface, where the cutoff bond distance was set to *R*_0_(O_W_–H_W_) = 1.2 Å with *p* = 6 and *q* = 6. The umbrella potentials were placed between 1.3 Å and 3.4 Å for CV1, and those of CV2 were chosen between 0.1 and 1.7, and the force constants of CV1 and CV2 were set to 20.7 eV Å^−2^ and 31.1 eV, respectively.

**Fig. 5:**
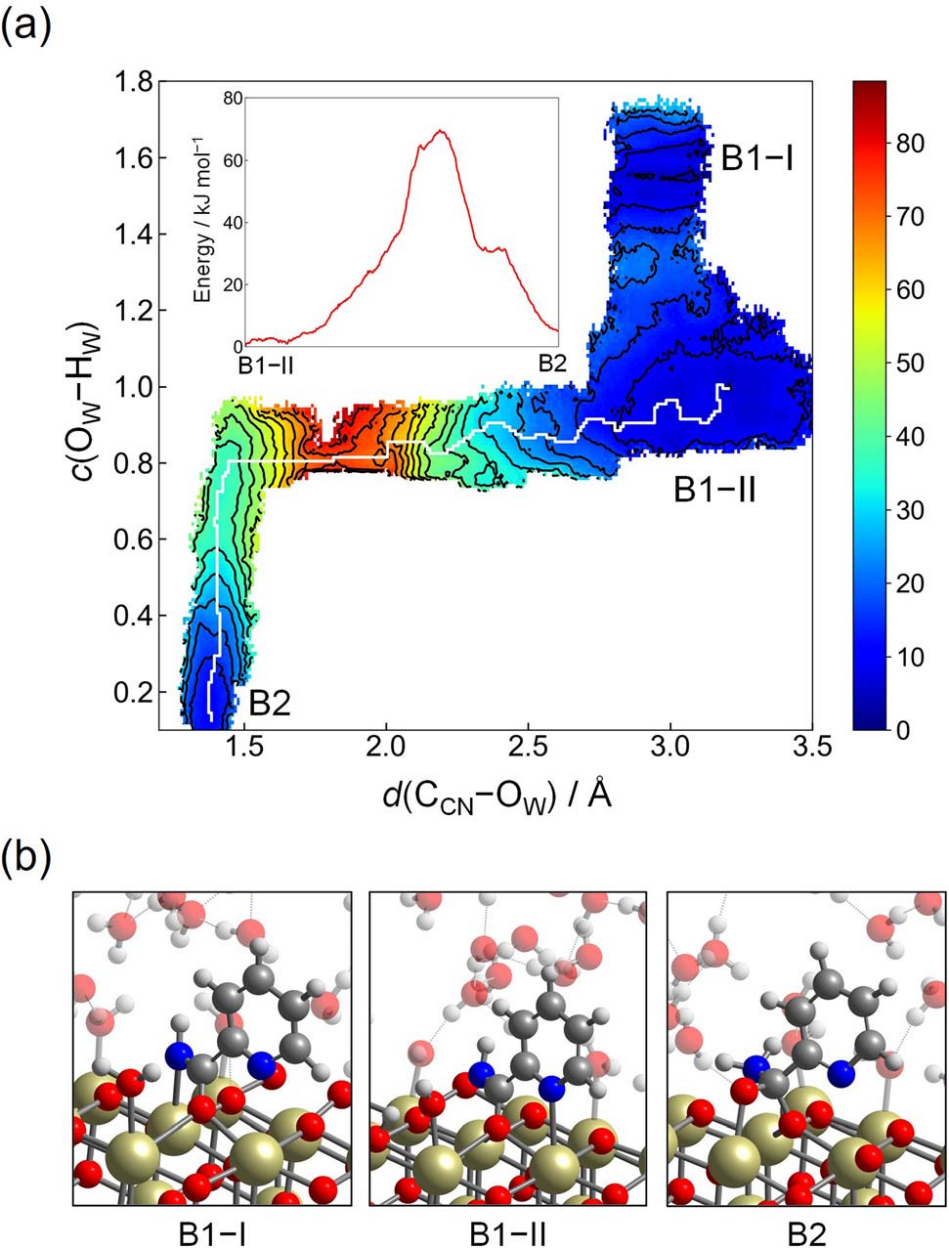
(a) Contour plot of the free energy surface (given in kJ mol^−1^) for the nucleophilic attack of a hydroxide ion to the C_CN_ atom (Step 2-(I)). The minimum free energy pathway, which is determined by the MULE algorithm, is shown in white. The inset shows the free energy profile along the minimum free energy pathway. (b) Snapshots of representative configuration near B1-I, B1-II, and B2.

Fig. 5(a) shows the contour plot of the reconstructed free energy surface along CV1 = *d*(C_CN_–O_W_) and CV2 = *c*(O_W_–H_W_), where the reactant region consists of the two stable states, B1-I (*d*(C_CN_–O_W_) ≈ 3.0 Å, *c*(O_W_–H_W_) ≈ 1.6) and B1-II (*d*(C_CN_–O_W_) ≈ 3.2 Å, *c*(O_W_–H_W_) ≈ 0.9). B1-I corresponds to the configuration where the attacking water molecule is coordinated to the nearby Ce atom (snapshot is given in Fig. 5(b)), while B1-II represents the configuration where the attacking water molecule is dissociatively adsorbed on the surface, being a hydroxide ion. The free energy profile between B1-I and B1-II simply represents the free energy difference between the water molecule and hydroxide ion on the nearby Ce atom. Free energy difference and activation energy between B1-I and B1-II are small, which indicates the facile proton hopping between the hydroxide ion and surface hydroxy group or adjacent water molecule, which has been already reported for the simulation of the water/CeO_2_(111) interface.^31^

**Fig. 6:**
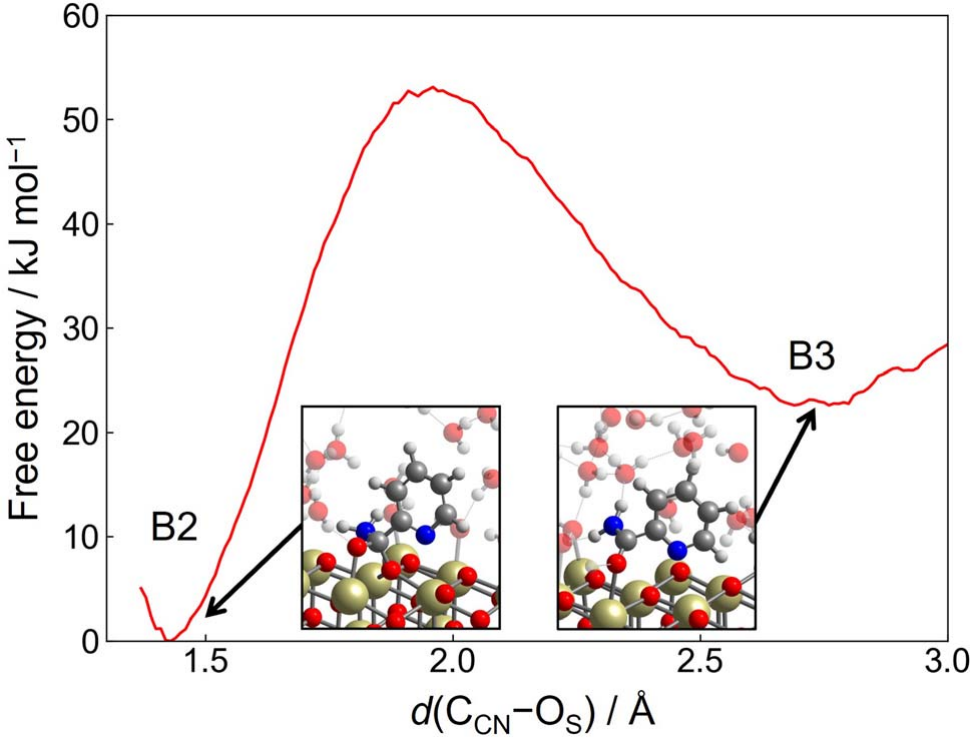
Free energy profile for the breaking of the C_CN_–O_S_ bond as a function of *d*(C_CN_–O_S_) starting from B2 in Step 2-(I).

Hydration reaction starts from B1-II, and the transition state region is found at *d*(C_CN_–O_W_) ≈ 1.8 Å and *c*(O_W_–H_W_) ≈ 0.8, representing the conformation of the nucleophilic attack of a hydroxide ion residing on the Ce atom to the C_CN_ atom. The free energy barrier is estimated as 70 kJ mol^−1^. After passing through this region, tautomerization readily occurs by a proton relay through the hydrogen bond network, leading to the amide product (picolinamide) (B2). The free energy barrier of 70 kJ mol^−1^ is appreciably lower than the value estimated in the hydration reaction in bulk water (120 kJ mol^−1^, see below and ESI†). This is rationalized by the unique adsorption structure of 2-cyanopyridine, where the C_CN_ atom is more positively charged due to the formation of protonated 2-cyanopyridine on the surface. At B2, there still exits the C_CN_–O_S_ bond, and the free energy profile for the breaking of the C_CN_–O_S_ bond was obtained by umbrella sampling using only one CV, *d*(C_CN_–O_S_). Fig. 6 shows the free energy profile for this step, and the free energy barrier of 53 kJ mol^−1^ was obtained. This barrier height is smaller than that of the nucleophilic attack of a hydroxide ion (B1-II → B2).

**Fig. 7:**
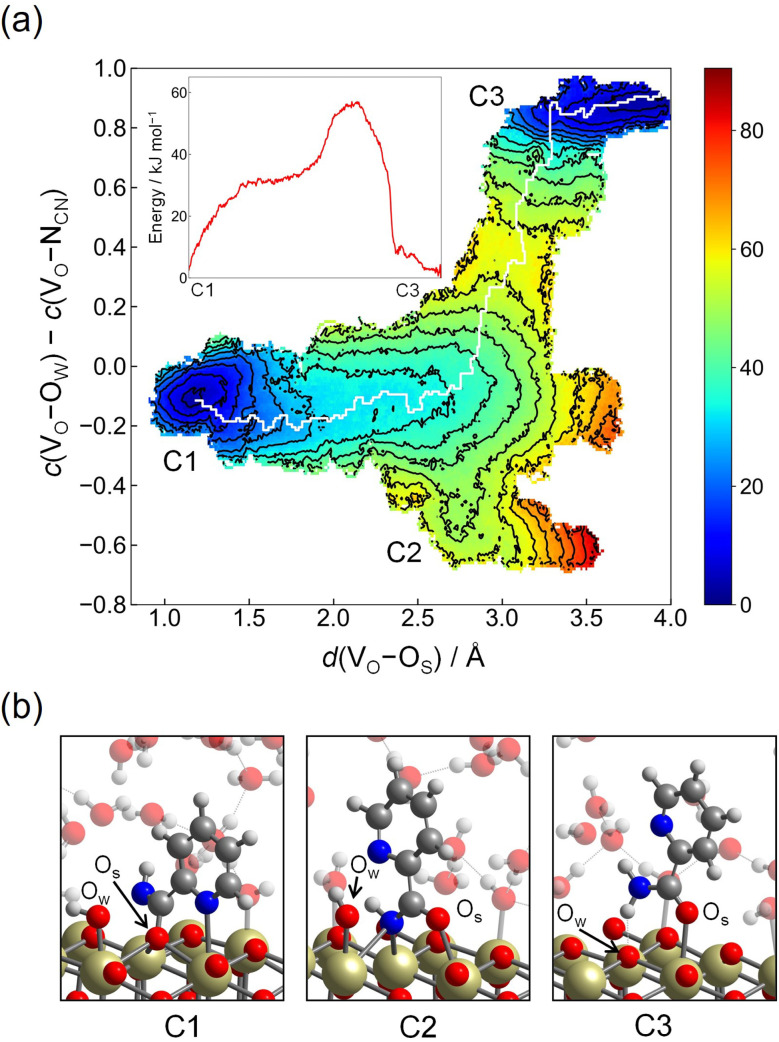
(a) Contour plot of the free energy surface (given in kJ mol^−1^) for the mechanism involving surface lattice oxygen atom (Step 2-(II)). The minimum free energy pathway, which is determined by the MULE algorithm, is shown in white. The inset shows the free energy profile along the minimum free energy pathway. (b) Snapshots of representative configuration near C1, C2, and C3. O_S_ represents the surface lattice oxygen involving the C_CN_–O_S_ bond and O_W_ represents an oxygen atom originating from solvent water molecule.

Next, we consider the second mechanism (Step 2-(II)), where the surface O_S_ atom is involved. In this step, the surface O_S_ atom of the C_CN_–O_S_ bond is introduced into 2-cyanopyridine, leading to the formation of picolinamide anion on the surface and an oxygen vacancy. Then, the resulting oxygen vacancy is replenished by a hydroxide ion on the surface. To investigate this reaction mechanism, the free energy surface is constructed by two CVs, CV1 and CV2. CV1 is the distance between the surface O_S_ atom of the C_CN_–O_S_ bond and the vacancy site V_O_ that is defined by the center of mass of the three surrounding surfaces Ce atoms, *d*(V_O_–O_S_). CV2 is defined as the difference between two coordination numbers, one of which is the coordination number between the vacancy site V_O_ and O atoms of solvent water molecules, *c*(V_O_–O_W_). The introduction of *c*(V_O_–O_W_) promotes the replenishment of the oxygen vacancy after the formation of picolinamide anion on the surface. The other one is defined as *c*(V_O_–N_CN_). We introduce this coordination number by noting that as the distance *d*(V_O_–O_S_) (=CV1) increases, the N_CN_ atom tends to fill the oxygen vacancy site due to the charge-negativity of the N_CN_ atom (see snapshot of C2 in Fig. 7(b)). The introduction of *c*(V_O_–N_CN_) is essential to promote the replenishment of the oxygen vacancy by a hydroxide ion rather than the N_CN_ atom. The cutoff bond distances of these coordination numbers were set to *R*_0_(V_O_–O_W_) = 2.0 Å with *p* = 6 and *q* = 4 and *R*_0_(V_O_–N_CN_) = 2.0 Å with *p* = 6 and *q* = 2, respectively.

Fig. 7(a) shows the contour plot of the free energy surface along CV1 = *d*(V_O_–O_S_) and CV2 = *c*(V_O_–O_W_) – *c*(V_O_–N_CN_). Starting from the minimum located at *d*(V_O_–O_S_) ≈ 1.2 Å and *c*(V_O_–O_W_) − *c*(V_O_–N_CN_) ≈ −0.1 (C1), which corresponds to the protonated adsorption structure of A3, the free energy increases slightly as the distance *d*(V_O_–O_S_) is increased until *d*(V_O_–O_S_) ≈ 2.6 Å. At *d*(V_O_–O_S_) ≈ 2.6 Å, the O_S_ atom is detached from the original lattice position, and the resulting configuration can be viewed as an adsorbed picolinamide anion near the oxygen vacancy site. From this structure, there are two pathways leading to C2 or C3. C2 is a metastable adsorption state of picolinamide anion with a shallow minimum at *d*(V_O_–O_S_) ≈ 2.7 Å and *c*(V_O_–O_W_) – *c*(V_O_–N_CN_) ≈ −0.5, where the N_CN_ atom of picolinamide anion partially fills the vacancy. When *d*(V_O_–O_S_) is increased further, there exists the barrier at *d*(V_O_–O_S_) ≈ 3.0 Å and *c*(V_O_–O_W_) – *c*(V_O_–N) ≈ 0.4 to accommodate a hydroxide ion at the vacancy site, which leads to C3. The barrier height associated with this process is estimated as 54 kJ mol^−1^, and it is lower than the value obtained in Step 2-(I) (70 kJ mol^−1^). After the vacancy site is filled by a hydroxide ion, the picolinamide anion readily accepts a proton from the surface hydroxy group that occupies the vacancy site, leading to the picolinamide product. The formed picolinamide is adsorbed on the surface by acid–base interaction between the O atom of the amide group and the surface Ce atom (see snapshot of C3 in Fig. 7(b)).

This reaction mechanism using surface lattice oxygen atom is quite unique in that the reaction occurs at room temperature. In CeO_2_-catalyzed reactions, it is generally accepted that the surface oxygen is involved in the redox reaction and that these reactions occur at much higher temperatures of ∼600 K.^4,58^ As an exception, it was reported that CeO_2_ works as an oxidation catalyst of alcohols at low temperatures around room temperature, where the surface lattice oxygen of CeO_2_ is used for the oxidation.^59^ When we monitor the oxidation state of Ce atoms for representative snapshots along the hydration reaction in Step 2-(II), the magnetic moment of all Ce atoms is almost zero, implying that the oxidation state of all Ce atoms is IV and the oxygen vacancy is accompanied by Ce(iv). It is suggested that Ce(iv) is maintained by the coordination of picolinamide anion and hydroxide ion, which are also stabilized by surrounding water molecules. The remarkable reactivity of CeO_2_ could be attributed to the flexibility of surface lattice oxygen atoms.

**Fig. 8:**
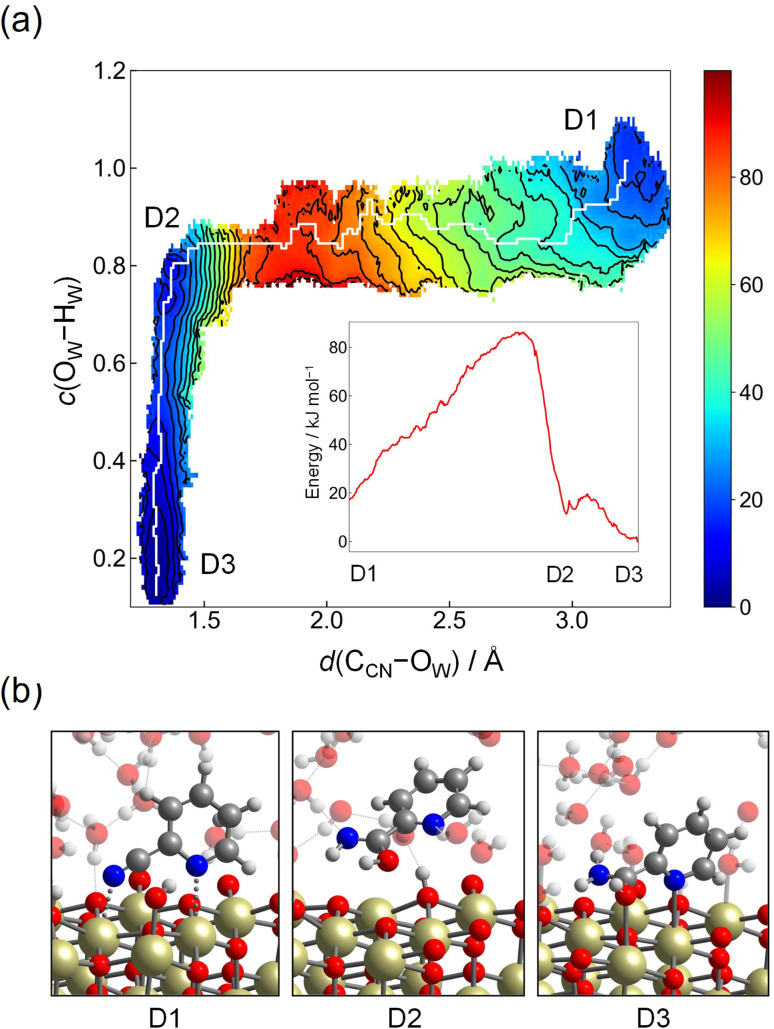
(a) Contour plot of the free energy surface (given in kJ mol^−1^) for the nucleophilic attack of a hydroxide ion to the C_CN_ atom from molecular adsorption structure (Step 2-(III)). The minimum free energy pathway, which is determined by the MULE algorithm, is shown in white. The inset shows the free energy profile along the minimum free energy pathway. (b) Snapshots of representative configuration near D1, D2, and D3.

As another possible reaction mechanism, hydration of 2-cyanopyridine from the molecular adsorption structure, which corresponds to St-II (see Fig. 2), is also examined in a similar manner to Step 2-(I), and this reaction mechanism is denoted as Step 2-(III) hereafter (see Fig. 3 for schematics). This mechanism is intuitively more plausible for nitrile hydration reaction on metal-oxide surface, where the CN group coordinated to the Lewis acid site of Ce atoms is nucleophilically attacked by a hydroxide ion on the surface, which is closely related to the reaction mechanism of nitrile hydration catalyzed by metal-complexes such as Ru-complexes.^60,61^ The starting configuration is the same as Step 1-(II) (corresponding to A1), and two CVs, *d*(C_CN_–O_W_) and *c*(O_W_–H_W_), are employed to obtain the free energy surface as has been chosen in Step 2-(I). The force constants of umbrella potential are set to be the same values as in Step 2-(I). The contour plot of the free energy surface spanned by *d*(C_CN_–O_W_) and *c*(O_W_–H_W_) is shown in Fig. 8(a). The reactant corresponds to D1, which is located at *d*(C_CN_–O_W_) ≈ 3.4 Å and *c*(O_W_–H_W_) ≈ 1.0, where an attacking hydroxide ion is coordinated on the nearby Ce atom. Here, the dissociation equilibrium of the attacking hydroxide ion and water molecule on the Ce atom is not considered as in Step 2-(I), which corresponds to the region of *c*(O_W_–H_W_) >1.0. As *d*(C_CN_–O_W_) becomes shorter, the molecule reaches the transition state region at *d*(C_CN_–O_W_) ≈ 1.7 Å and *c*(O_W_–H_W_) ≈ 0.8, where the free energy barrier is calculated to be 69 kJ mol^−1^. After passing through the transition state region, the shallow minimum is seen at *d*(C_CN_–O_W_) ≈ 1.3 Å and *c*(O_W_–H_W_) ≈ 0.8 (D2), which corresponds to an imidic acid. Then, picolinamide (D3) is formed by tautomerization through a proton relay. The free energy barrier of 69 kJ mol^−1^ is comparable to that of Step 2-(I) (70 kJ mol^−1^), but the high stability of the protonated adsorption state of A3, compared to molecular adsorption state of A1 (difference in free energy is 76 kJ mol^−1^), would eliminate the possibility of taking this reaction pathway. The hydration of 2-cyanopyridine in the bulk water is also examined for reference (the details are provided in the ESI†), and in this case, the free energy barrier was estimated as 120 kJ mol^−1^, which is significantly high compared to the reaction occurring at the interface of water/CeO_2_. In the experiments of CeO_2_-catalyzed reaction, the activation energy was estimated to be 81.7 kJ mol^−1^ and also the activation Gibbs energy was reported to be 79.0 kJ mol^−1^ by the Eyring plot.^19^ These values are slightly higher than the barrier heights of Step 2-(I) (70 kJ mol^−1^) and Step 2-(II) (54 kJ mol^−1^) estimated in the simulation. One reason would be the simulation temperature of 360 K in our simulations. We employ this temperature because it is a good compromise for describing the structural properties of ambient liquid water for PBE and its derived functionals in DFT calculations.^62,63^

**Fig. 9:**
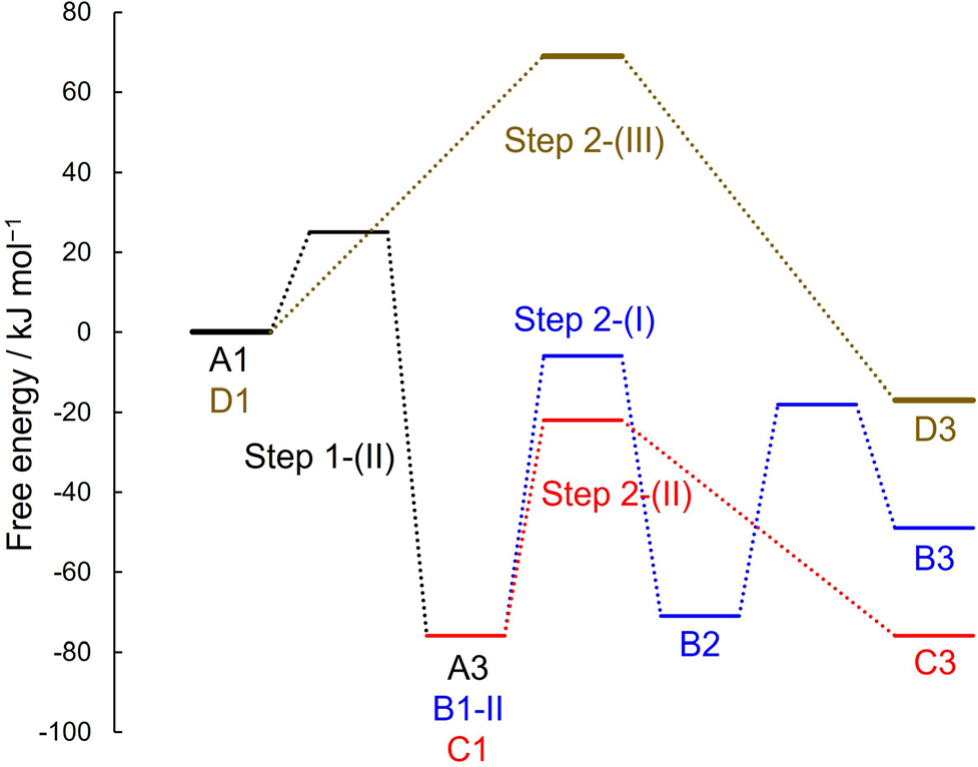
Free energy diagram starting from molecular adsorption state of A1 and D1, where zero of the free energy is set to this state.

Fig. 9 depicts the free energy diagram for the reaction pathways investigated in this work. Starting from the molecular adsorption state (A1), a protonated species with the C_CN_–O_S_ covalent bond is formed (A3). From this strong adsorption state, two reaction pathways are possible. Based on the difference in the free energy barrier, the reaction involving surface lattice oxygen is energetically more favorable.

### 3.3. Experimental verification

If the reaction mechanism involving surface lattice oxygen (Step 2-(II)) is the main reaction pathway, the lattice oxygen will be introduced into 2-cyanopyridine to form picolinamide in the hydration of 2-cyanopyridine. If not, the oxygen atoms of water molecules will be introduced into 2-cyanopyridine. We conducted two experiments: using (i) CeO_2_ with lattice oxygen partially substituted with ^18^O (^18^O-substituted CeO_2_) and (ii) ^18^O-substituted water (H_2_^18^O) solvent. The introduction of ^18^O in picolinamide can be confirmed by the difference in the mass number of picolinamide; The mass number of the product with ^16^O and ^18^O are 122 and 124, respectively. The experimental details are provided in ESI.†

**Fig. 10:**
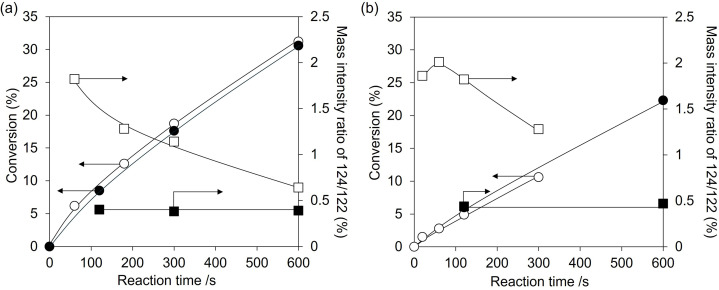
Conversion and mass intensity ratio of 124/122 in hydration of 2-cyanopyridine with ^18^O-substituted CeO_2_ and CeO_2_ catalysts at (a) 279 K and (b) 274 K. Marks of ^18^O-substituted CeO_2_. Conversion: white circle, mass intensity ratio of 124/122: white square. Marks of CeO_2_. Conversion: black circle, mass intensity ratio of 124/122: black square. Reaction conditions (a): CeO_2_ or ^18^O-substituted CeO_2_ 100 mg (0.58 mmol), 2-cyanopyridine 104 mg (1.0 mmol), H_2_O 5 g, 279 K. Reaction conditions (b): CeO_2_ or ^18^O-substituted CeO_2_ 100 mg (0.58 mmol), 2-cyanopyridine 104 mg (1.0 mmol), H_2_O 5 g, 274 K. The detailed data are shown in Table S1.†

**Fig. 11:**
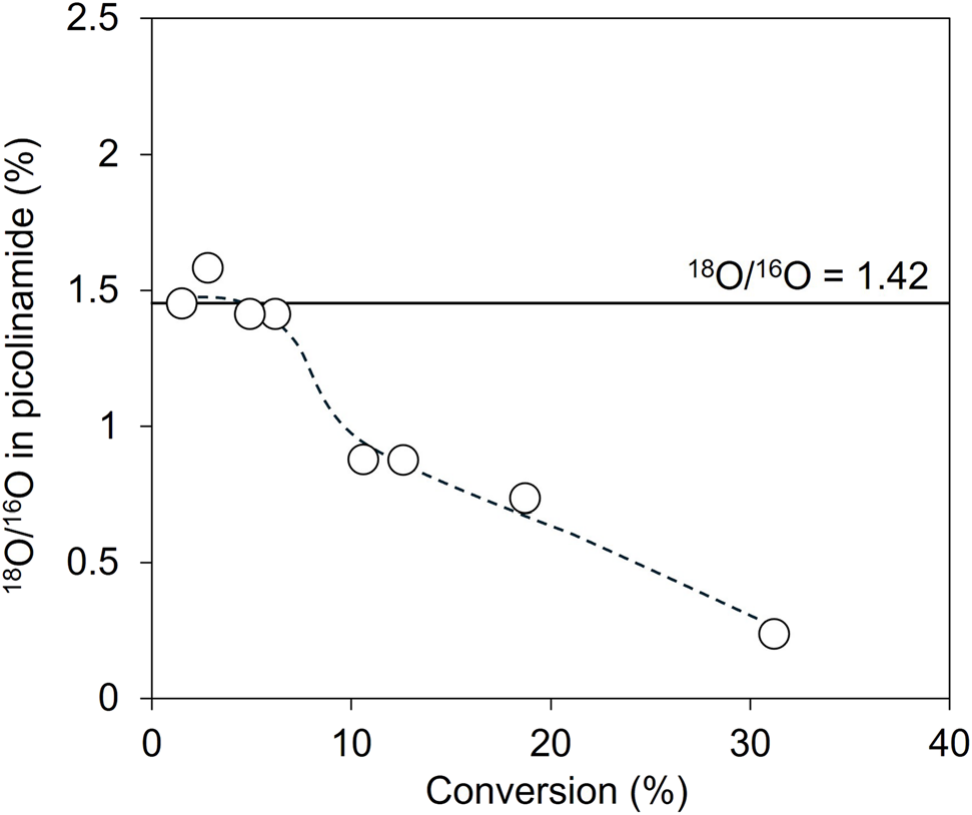
The relationship between the ^18^O/^16^O in picolinamide and the conversion in hydration of 2-cyanopyridine over ^18^O-substituted CeO_2_. The detailed data are shown in Table S2.†

In the first experiment, we prepared ^18^O-substituted CeO_2_ by introducing ^18^O_2_ after hydrogen reduction at 873 K, and the substituted ratio of ^18^O/^16^O in ^18^O-introduced CeO_2_ was determined to be 1.42% by O_2_ adsorption of the reduced CeO_2_ (Fig. S4†). Hydration of 2-cyanopyridine was conducted at 274 K and 279 K by using the ^18^O-introduced CeO_2_, and the product was confirmed by mass spectroscopy. Fig. 10 shows the time course of the hydration of 2-cyanopyridine with CeO_2_ and ^18^O-substituted CeO_2_. In the initial stage of the reaction, the mass intensity ratio of 124/122 in picolinamide with ^18^O-substituted CeO_2_ was significantly higher (about five times) compared to that with CeO_2_, confirming that ^18^O from ^18^O-substituted CeO_2_ was introduced into the picolinamide. The relationship between the ^18^O/^16^O in picolinamide and conversion is shown in Fig. 11. As a result, the ^18^O/^16^O was around 1.4% at low conversions, which is in good agreement with the ^18^O/^16^O in ^18^O-substituted CeO_2_ (1.42%), and the ratio decreased as the conversion increased. This result can be interpreted as follows. In the initial stage of the reaction, picolinamide was produced with the surface lattice oxygen of ^18^O-substituted CeO_2,_ and therefore, the ^18^O/^16^O in picolinamide (∼1.41%) is almost equal to that of the ^18^O-substituted CeO_2_ (1.42%). As the reaction consumes ^18^O in the ^18^O-substituted CeO_2_, ^16^O is replenished from water and the surface ^18^O/^16^O of the ^18^O-substituted CeO_2_ decreased, resulting in the decrease in the ^18^O/^16^O in picolinamide. If the reaction mechanism of Step 2-(I) is involved, ^16^O of H_2_O will be introduced into the product and ^18^O/^16^O in picolinamide would be lower than 1.42%.

**Fig. 12:**
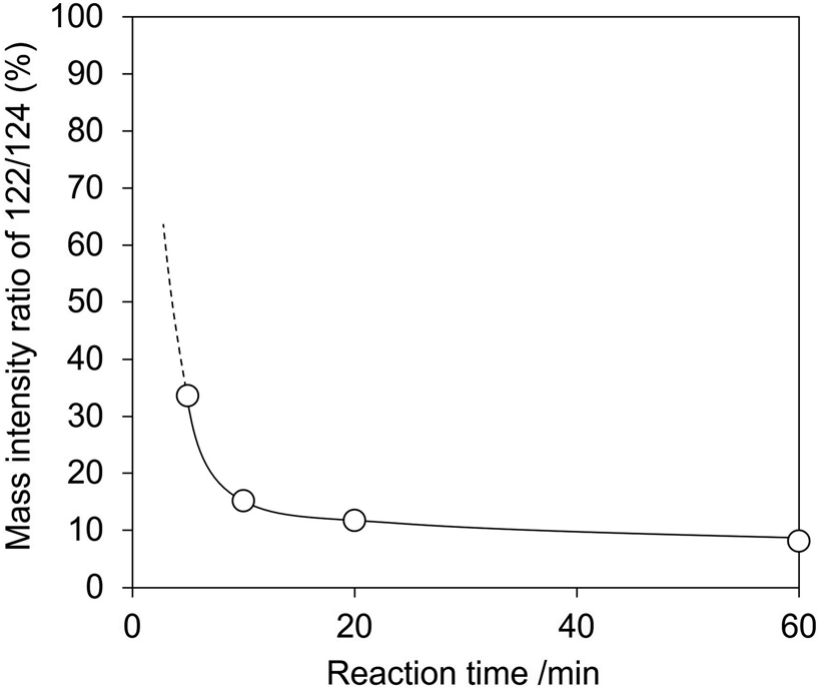
Mass intensity ratio of 122/124 in hydration of 2-cyanopyridine in H_2_^18^O over CeO_2_. Reaction conditions: CeO_2_ 100 mg (0.58 mmol), 2-cyanopyridine 104 mg (1.0 mmol), H_2_O or H_2_^18^O 3 g, 278 K.

In the second experiment, we conducted the hydration of 2-cyanopyridine with H_2_^18^O solvent and CeO_2_. If picolinamide is produced by the surface lattice oxygen of CeO_2_, the mass number of the produced picolinamide is 122. On the other hand, if picolinamide is produced directly by water molecules, the mass number of the produced picolinamide is 124. At first, we checked the reactivity of the hydration of 2-cyanopyridine with H_2_O and H_2_^18^O (Fig. S5 and Table S3†), showing no significant difference between the reactions. A typical example of the mass spectrum of the produced picolinamide is shown in Fig. S6,† and the results are shown in Fig. 12 and Table S3.† The mass intensity ratio of 122/124 increased with decreasing the reaction time, and the mass number ratio at 0 min is expected to be quite high, indicating that under the conditions where TON is 1 for the surface lattice oxygen of CeO_2_, a quantitative amount of the amide with ^16^O, namely with a molecular weight of 122, was formed. Therefore, the hydration of 2-cyanopyridine proceeds with the involvement of surface lattice oxygen, and these two experimental results strongly support the reaction mechanism suggested by the DFT-MD simulations.

## 4. Conclusions

The detailed mechanism of the hydration of 2-cyanopyridine over CeO_2_ catalysts was investigated by the DFT-MD simulations. The possible reaction mechanism was discussed by depicting the free energy landscape. The adsorption of 2-cyanopyridine onto CeO_2_(111) is facile, leading to the stable adsorption structure of the protonated species, which is covalently bonded to the surface lattice oxygen atom. Two reaction schemes were compared from this adsorption structure, and the free energy calculations reveal that the reaction mechanism involving the surface lattice oxygen was energetically preferable with the energy barrier associated with this process being 54 kJ mol^−1^. The other possible mechanism is the nucleophilic attack of a hydroxide ion formed on the surface Ce atom to the C atom of the protonated nitrile species. The free energy barrier associated with this process is calculated to be 70 kJ mol^−1^, which is higher than the former process. The former lattice oxygen insertion mechanism is quite unique considering that this reaction proceeds readily at room temperature or below. The contribution of the surface lattice oxygen to the hydration reaction was also investigated by the two experiments using H_2_^18^O solvent and ^18^O-exchanged CeO_2_ catalyst. It was confirmed that the lattice oxygen-introduced product was obtained by both experiments, which strongly supports theoretical prediction. In a related study, the oxygen vacancy-assisted mechanism over CeO_2_ catalyst was reported in the direct dimethyl carbonate synthesis from CO_2_ and methanol.^64^ The flexibility of surface oxygen and the unique acid–base properties of CeO_2_, which can accommodate substrate with the covalent bond along with acid–base interactions and activate water molecules on the surface, are the key factors in its high reactivity for various organic reactions. These unique characteristics of CeO_2_ will be further utilized in the development of more efficient and environmentally friendly catalysts for various organic reactions at low temperatures. In particular, this unique reactivity of the lattice oxygen of CeO_2_ in water is believed to be effective for hydrolysis and hydration reactions using water, making it promising not only for green reaction systems that employ water as a green solvent, but also for applications in the low-temperature hydrolysis of plastics such as polyesters and polyamides, which have recently garnered attention.

## Author contributions

T. E. and A. N. performed calculations. Y. K. and M. T. conducted experiments. M. T. and A. N. conceived of the work. All authors contributed to the writing and proofing of the final manuscript.

## Conflicts of interest

There are no conflicts to declare.

## Supplementary Material

SC-016-D4SC06294A-s001

## Data Availability

Additional data supporting this article are included in the ESI.†

## References

[cit1] Park S., Vohs J. M., Gorte R. J. (2000). Direct Oxidation of Hydrocarbons in a Solid-Oxide Fuel Cell. Nature.

[cit2] Fu Q., Saltsburg H., Flytzani-Stephanopoulos M. (2003). Active Nonmetallic Au and Pt Species on Ceria-Based Water-Gas Shift Catalysts. Science.

[cit3] Deluga G. A., Salge J. R., Schmidt L. D., Verykios X. E. (2004). Renewable Hydrogen from Ethanol by Autothermal Reforming. Science.

[cit4] Montini T., Melchionna M., Monai M., Fornasiero P. (2016). Fundamentals and Catalytic Applications of CeO_2_-Based Materials. Chem. Rev..

[cit5] Mullins D. R. (2015). The Surface Chemistry of Cerium Oxide. Surf. Sci. Rep..

[cit6] Ganduglia-Pirovano M. V. (2015). The Non-Innocent Role of Cerium Oxide in Heterogeneous Catalysis: A Theoretical Perspective. Catal. Today.

[cit7] Campbell C. T. (2012). Electronic Perturbations. Nat. Chem..

[cit8] Bruix A., Rodriguez J. A., Ramírez P. J., Senanayake S. D., Evans J., Park J. B., Stacchiola D., Liu P., Hrbek J., Illas F. (2012). A New Type of Strong Metal–Support Interaction and the Production of H_2_ through the Transformation of Water on Pt/CeO_2_(111) and Pt/CeO_x_/TiO_2_(110) Catalysts. J. Am. Chem. Soc..

[cit9] Lykhach Y., Kozlov S. M., Skála T., Tovt A., Stetsovych V., Tsud N., Dvořák F., Johánek V., Neitzel A., Mysliveček J., Fabris S., Matolín V., Neyman K. M., Libuda J. (2016). Counting Electrons on Supported Nanoparticles. Nat. Mater..

[cit10] Ota N., Tamura M., Nakagawa Y., Okumura K., Tomishige K. (2015). Hydrodeoxygenation of Vicinal OH Groups over Heterogeneous Rhenium Catalyst Promoted by Palladium and Ceria Support. Angew. Chem., Int. Ed..

[cit11] Ota N., Tamura M., Nakagawa Y., Okumura K., Tomishige K. (2016). Performance, Structure, and Mechanism of ReO_x_–Pd/CeO_2_ Catalyst for Simultaneous Removal of Vicinal OH Groups with H_2_. ACS Catal..

[cit12] Tazawa S., Ota N., Tamura M., Nakagawa Y., Okumura K., Tomishige K. (2016). Deoxydehydration with Molecular Hydrogen over Ceria-Supported Rhenium Catalyst with Gold Promoter. ACS Catal..

[cit13] Cao J., Tamura M., Hosaka R., Nakayama A., Hasegawa J., Nakagawa Y., Tomishige K. (2020). Mechanistic Study on Deoxydehydration and Hydrogenation of Methyl Glycosides to Dideoxy Sugars over a ReO_x_–Pd/CeO_2_ Catalyst. ACS Catal..

[cit14] Tamura M., Hayashigami N., Nakayama A., Nakagawa Y., Tomishige K. (2022). Heterogeneous Enantioselective Hydrogenation of Ketones by 2-Amino-2’-Hydroxy-1,1’binaphthyl-Modified CeO_2_-Supported Ir Nanoclusters. ACS Catal..

[cit15] Vivier L., Duprez D. (2010). Ceria-Based Solid Catalysts for Organic Chemistry. ChemSusChem.

[cit16] Sato S., Sato F., Gotoh H., Yamada Y. (2013). Selective Dehydration of Alkanediols into Unsaturated Alcohols over Rare Earth Oxide Catalysts. ACS Catal..

[cit17] Tamura M., Shimizu K., Satsuma A. (2012). CeO_2_-Catalyzed Transformations of Nitriles and Amides. Chem. Lett..

[cit18] Tamura M., Satsuma A., Shimizu K. (2013). CeO_2_-Catalyzed Nitrile Hydration to Amide: reaction Mechanism and Active Sites. Catal. Sci. Technol..

[cit19] Tamura M., Sawabe K., Tomishige K., Satsuma A., Shimizu K. (2015). Substrate-Specific Heterogeneous Catalysis of CeO_2_ by Entropic Effects *via* Multiple Interactions. ACS Catal..

[cit20] Honda M., Tamura M., Nakagawa Y., Sonehara S., Suzuki K., Fujimoto K., Tomishige K. (2013). Ceria-Catalyzed Conversion of Carbon Dioxide into Dimethyl Carbonate with 2-Cyanopyridine. ChemSusChem.

[cit21] Tomishige K., Tamura M., Nakagawa Y. (2019). CO_2_ Conversion with Alcohols and Amines into Carbonates, Ureas, and Carbamates over CeO_2_ Catalyst in the Presence and Absence of 2-Cyanopyridine. Chem. Rec..

[cit22] Tamura M., Nakagawa Y., Tomishige K. (2022). Direct CO_2_ Transformation to Aliphatic Polycarbonates. Asian J. Org. Chem..

[cit23] Tamura M., Kishi R., Nakayama A., Nakagawa Y., Hasegawa J., Tomishige K. (2017). Formation of a New, Strongly Basic Nitrogen Anion by Metal Oxide Modification. J. Am. Chem. Soc..

[cit24] Tamura M., Hiwatashi D., Gu Y., Nakayama A., Nakagawa Y., Tomishige K. (2021). Organic Compound Modification of CeO_2_ and 2-Cyanopyridine Hybrid Catalyst in Carbonate Synthesis from CO_2_ and Alcohols. J. CO_2_ Util..

[cit25] Tamura M., Sagawa E., Nakayama A., Nakagawa Y., Tomishige K. (2021). Hydrogen Atom Abstraction by Heterogeneous–Homogeneous Hybrid Catalyst of CeO_2_ and 2-Cyanopyridine *via* Redox of CeO_2_ for C–H Bond Oxidation with Air. ACS Catal..

[cit26] Sun W., Li P., Yabushita M., Nakagawa Y., Wang Y., Nakayama A., Tomishige K. (2023). Comparative Study between 2-Furonitrile and 2-Cyanopyridine as Dehydrants in Direct Synthesis of Dialkyl Carbonates from CO_2_ and Alcohols over Cerium Oxide Catalyst. ChemSusChem.

[cit27] Seki T., Takeda K., Nakayama A. (2022). Ab Initio Molecular Dynamics Simulations for Adsorption Structures of Picolinic Acid at the Water/CeO_2_ Interface. J. Phys. Chem. C.

[cit28] Farnesi Camellone M., Negreiros Ribeiro F., Szabová L., Tateyama Y., Fabris S. (2016). Catalytic Proton Dynamics at the Water/Solid Interface of Ceria-Supported Pt Clusters. J. Am. Chem. Soc..

[cit29] Ren Z., Liu N., Chen B., Li J., Mei D. (2018). Theoretical Investigation of the Structural Stabilities of Ceria Surfaces and Supported Metal Nanocluster in Vapor and Aqueous Phases. J. Phys. Chem. C.

[cit30] Agosta L., Arismendi-Arrieta D., Dzugutov M., Hermansson K. (2023). Origin of the Hydrophobic Behaviour of Hydrophilic CeO_2_. Angew. Chem., Int. Ed..

[cit31] Kobayashi T., Ikeda T., Nakayama A. (2024). Long-Range Proton and Hydroxide Ion Transfer Dynamics
at the Water/CeO_2_ Interface in the Nanosecond Regime: Reactive Molecular Dynamics Simulations and Kinetic Analysis. Chem. Sci..

[cit32] Kühne T. D., Iannuzzi M., Del Ben M., Rybkin V. V., Seewald P., Stein F., Laino T., Khaliullin R. Z., Schütt O., Schiffmann F., Golze D., Wilhelm J., Chulkov S., Bani-Hashemian M. H., Weber V., Borštnik U., Taillefumier M., Jakobovits A. S., Lazzaro A., Pabst H., Müller T., Schade R., Guidon M., Andermatt S., Holmberg N., Schenter G. K., Hehn A., Bussy A., Belleflamme F., Tabacchi G., Glöβ A., Lass M., Bethune I., Mundy C. J., Plessl C., Watkins M., VandeVondele J., Krack M., Hutter J. (2020). CP2K: An Electronic Structure and Molecular Dynamics Software Package - Quickstep: Efficient and Accurate Electronic Structure Calculations. J. Chem. Phys..

[cit33] VandeVondele J., Hutter J. (2007). Gaussian Basis Sets for Accurate Calculations on Molecular Systems in Gas and Condensed Phases. J. Chem. Phys..

[cit34] Goedecker S., Teter M., Hutter J. (1996). Separable Dual-Space Gaussian Pseudopotentials. Phys. Rev. B:Condens. Matter Mater. Phys..

[cit35] Hartwigsen C., Goedecker S., Hutter J. (1998). Relativistic Separable Dual-Space Gaussian Pseudopotentials from H to Rn. Phys. Rev. B:Condens. Matter Mater. Phys..

[cit36] Wang Y.-G., Mei D., Li J., Rousseau R. (2013). DFT + *U* Study on the Localized Electronic States and Their Potential Role During H_2_O Dissociation and CO Oxidation Processes on CeO_2_(111) Surface. J. Phys. Chem. C.

[cit37] Esch F., Fabris S., Zhou L., Montini T., Africh C., Fornasiero P., Comelli G., Rosei R. (2005). Electron Localization Determines Defect Formation on Ceria Substrates. Science.

[cit38] Paier J., Penschke C., Sauer J. (2013). Oxygen Defects and Surface Chemistry of Ceria: Quantum Chemical Studies Compared to Experiment. Chem. Rev..

[cit39] Nolan M., Verdugo V. S., Metiu H. (2008). Vacancy Formation and CO Adsorption on Gold-Doped Ceria Surfaces. Surf. Sci..

[cit40] Nörenberg H., Briggs G. A. D. (1999). Defect Formation on CeO_2_(111) Surfaces after Annealing Studied by STM. Surf. Sci..

[cit41] Nolan M., Parker S. C., Watson G. W. (2005). The Electronic Structure of Oxygen Vacancy Defects at the Low Index Surfaces of Ceria. Surf. Sci..

[cit42] Huang X., Zhang K., Peng B., Wang G., Muhler M., Wang F. (2021). Ceria-Based Materials for Thermocatalytic and Photocatalytic Organic Synthesis. ACS Catal..

[cit43] Tian L., Liao Y.-S., Xiao Z., Sun G., Chou J.-P., Wong C.-Y., Ho J. C., Zhao Y., Chou P.-T., Peng Y.-K. (2024). Unveiling the Pivotal Role of Ce Coordination Structures and Their Surface Arrangements in Governing 2-Cyanopyridine Hydrolysis for Direct Dimethyl Carbonate Synthesis from CO_2_ and Methanol. ACS Catal..

[cit44] Gerward L., Olsen J. S. (1993). Powder Diffraction Analysis of Cerium Dioxide at High Pressure. Powder Diffr..

[cit45] Nakajima A., Yoshihara A., Ishigame M. (1994). Defect-Induced Raman Spectra in Doped CeO_2_. Phys. Rev. B:Condens. Matter Mater. Phys..

[cit46] Tuckerman M. E. , Statistical Mechanics, Oxford Graduate Texts, Oxford University Press, Incorporated, Oxford, 2nd edn, 2023

[cit47] Laio A., Parrinello M. (2002). Escaping Free-Energy Minima. Proc. Natl. Acad. Sci. U.S.A..

[cit48] Barducci A., Bussi G., Parrinello M. (2008). Well-Tempered Metadynamics: A Smoothly Converging and Tunable Free-Energy Method. Phys. Rev. Lett..

[cit49] Torrie G. M., Valleau J. P. (1974). Monte Carlo Free Energy Estimates Using Non-Boltzmann Sampling: Application to the Sub-Critical Lennard-Jones Fluid. Chem. Phys. Lett..

[cit50] Wang F., Landau D. P. (2001). Efficient, Multiple-Range Random Walk Algorithm to Calculate the Density of States. Phys. Rev. Lett..

[cit51] Yang Y. I., Zhang J., Che X., Yang L., Gao Y. Q. (2016). Efficient Sampling over Rough Energy Landscapes with High Barriers: A Combination of Metadynamics with Integrated Tempering Sampling. J. Chem. Phys..

[cit52] Comer J., Gumbart J. C., Hénin J., Lelièvre T., Pohorille A., Chipot C. (2015). The Adaptive Biasing Force Method: Everything You Always Wanted To Know but Were Afraid To Ask. J. Phys. Chem. B.

[cit53] Abraham M. J., Murtola T., Schulz R., Páll S., Smith J. C., Hess B., Lindahl E. (2015). GROMACS: High Performance Molecular Simulations through Multi-Level Parallelism from Laptops to Supercomputers. SoftwareX.

[cit54] Kumar S., Rosenberg J. M., Bouzida D., Swendsen R. H., Kollman P. A. (1992). The Weighted Histogram Analysis Method for Free-energy Calculations on Biomolecules. I. The Method. J. Comput. Chem..

[cit55] Grossfield A. , WHAM: The Weighted Histogram Analysis Method, Version 2.0.11, http://membrane.urmc.rochester.edu/?page_id=126

[cit56] Tribello G. A., Bonomi M., Branduardi D., Camilloni C., Bussi G. (2014). PLUMED 2: New Feathers for an Old Bird. Comput. Phys. Commun..

[cit57] Fu H., Chen H., Wang X., Chai H., Shao X., Cai W., Chipot C. (2020). Finding an Optimal Pathway on a Multidimensional Free-Energy Landscape. J. Chem. Inf. Model..

[cit58] Vincent J. L., Crozier P. A. (2021). Atomic Level Fluxional Behavior and Activity of CeO_2_-Supported Pt Catalysts for CO Oxidation. Nat. Commun..

[cit59] Tamura M., Tomishige K. (2015). Redox Properties of CeO_2_ at Low Temperature: The Direct Synthesis of Imines from Alcohol and Amine. Angew. Chem., Int. Ed..

[cit60] Guo B., De Vries J. G., Otten E. (2019). Hydration of Nitriles Using a Metal–Ligand Cooperative Ruthenium Pincer Catalyst. Chem. Sci..

[cit61] Yadav S., Gupta R. (2022). Hydration of Nitriles Catalyzed by Ruthenium Complexes: Role of Dihydrogen Bonding Interactions in Promoting Base-Free Catalysis. Inorg. Chem..

[cit62] Prendergast D., Grossman J. C., Galli G. (2005). The Electronic Structure of Liquid Water within Density-Functional Theory. J. Chem. Phys..

[cit63] Gillan M. J., Alfè D., Michaelides A. (2016). Perspective: How Good Is DFT for Water?. J. Chem. Phys..

[cit64] Stoian D., Sugiyama T., Bansode A., Medina F., Van Beek W., Hasegawa J., Nakayama A., Urakawa A. (2023). Dimethyl Carbonate Synthesis from CO_2_ and Methanol over CeO_2_: Elucidating the Surface Intermediates and Oxygen Vacancy-Assisted Reaction Mechanism. Chem. Sci..

