# Supplementary material for: Lattice oxygen insertion mechanism in CeO2-catalyzed reactions in water: nitrile hydration reaction
Source: Chem Sci. 2024 Dec 2;16(2):939–51. doi: 10.1039/d4sc06294a (PMC11627105; doi:10.1039/d4sc06294a)
Supplement: SC-016-D4SC06294A-s001 [file SC-016-D4SC06294A-s001.pdf]

Supplementary Information (SI) for Chemical Science.
This journal is © The Royal Society of Chemistry 2024

Supporting Information

Lattice Oxygen Insertion Mechanism in CeO_2-Catalyzed Reactions in Water: Nitrile Hydration Reaction

Takaaki Endo,[1] Tatsushi Ikeda,[1] Koki Muraoka,[1] Yusuke Kita,[2] Masazumi Tamura,[2,*] and Akira Nakayama[1,*]

[1]Department of Chemical System Engineering, The University of Tokyo, Tokyo 113-8656, Japan
[2]Department of Chemistry and Bioengineering, School of Engineering, Osaka Metropolitan University,
3-3-138, Sugimoto, Sumiyoshi-ku, Osaka 558-8585, Japan

Corresponding Authors*: mtamura@omu.ac.jp, nakayama@chemsys.t.u-tokyo.ac.jp

Substrate Specificity: Comparison with 4-cyanopyridine

We examine the substrate specificity of this unique hydration reaction by comparing it with 4-cyanopyridine. As experimentally shown before, the reaction rate of hydration of 4-cyanopyridine is 10^{-7}-order of magnitude smaller than that of 2-cyanopyridine, and our previous calculations showed that the C_{CN}−O_S bond formation is hindered by the steric interaction between the pyridine ring and the surface.[1] The adsorption structures of 4-cyanopyridine over CeO_2(111) are shown in Figure S1, where the three structures, each corresponding to the adsorption structures of 2-cyanopyridine given in Figure 2, are provided. The structure with C_{CN}−O_S bond (**St−I** in Figure S1(a)) exhibits weak adsorption with an adsorption energy of only −3.7 kJ/mol, where the surface oxygen atom (O_S) is dislocated from the surface due to the steric hindrance between the pyridine ring and the surface. The other two adsorption structures have similar adsorption energies to those of 2-cyanopyridine.

To demonstrate the difference in stability between 2-cyanoyridine and 4-cyanopyridine in water environment, the free energy profile for the adsorption process of 4-cyanopyrdine is examined. The CVs were set to the same as those for 2-cyanopyridine, and the two-dimensional contour plot of the free energy surface along CV1 = $d(C_{CN}-O_S)$ and CV2 = $c(N_{CN}-H_W)$ is shown in Figure S2. The final product is the protonated 4-cyanopyridine, and as clearly seen, the free energy barrier leading to the product state (**F3**) is 68 kJ/mol, which is much higher than that of 2-cyanopyridine (25 kJ/mol). This unfavorable adsorption of 4-cyanopyridine is related to the very low activity of the hydration reaction.

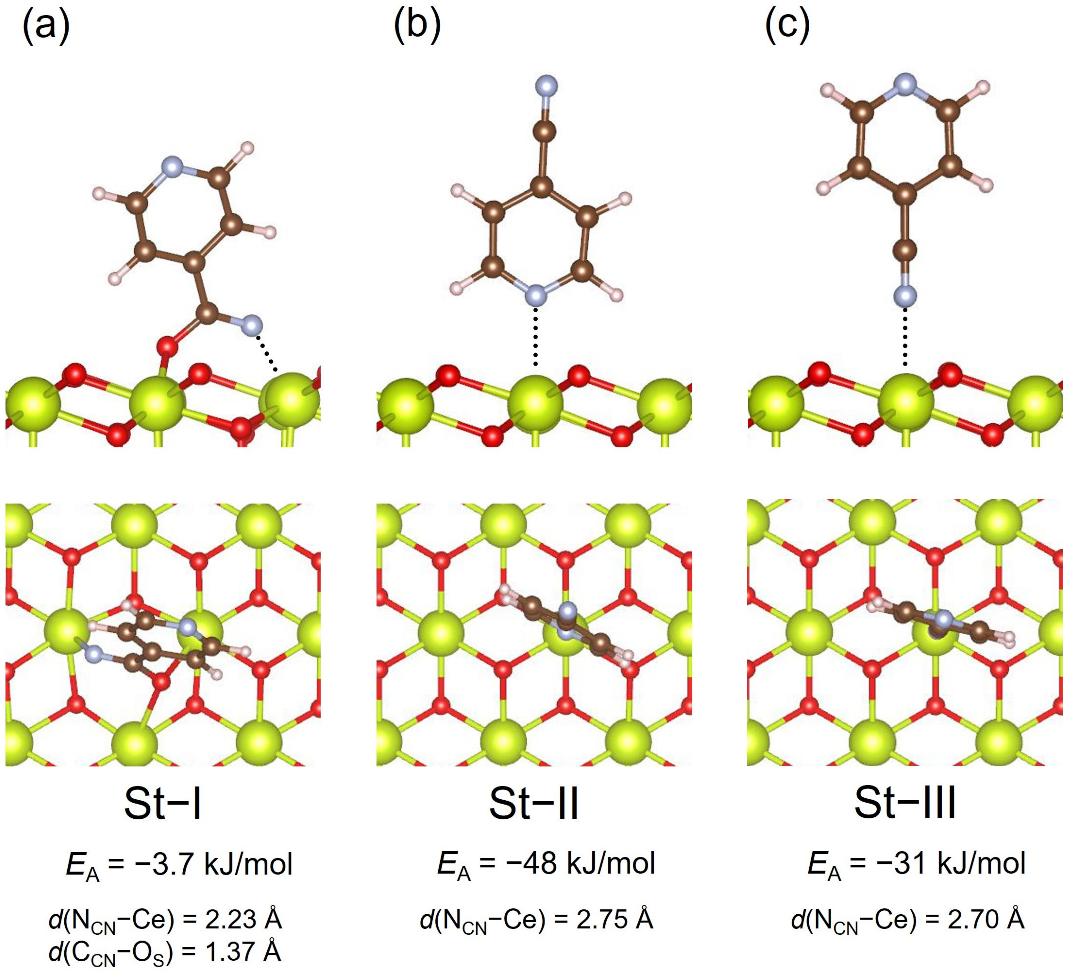

Figure S1. Adsorption structures of 4-cyanopyridine on $CeO_2(111)$.

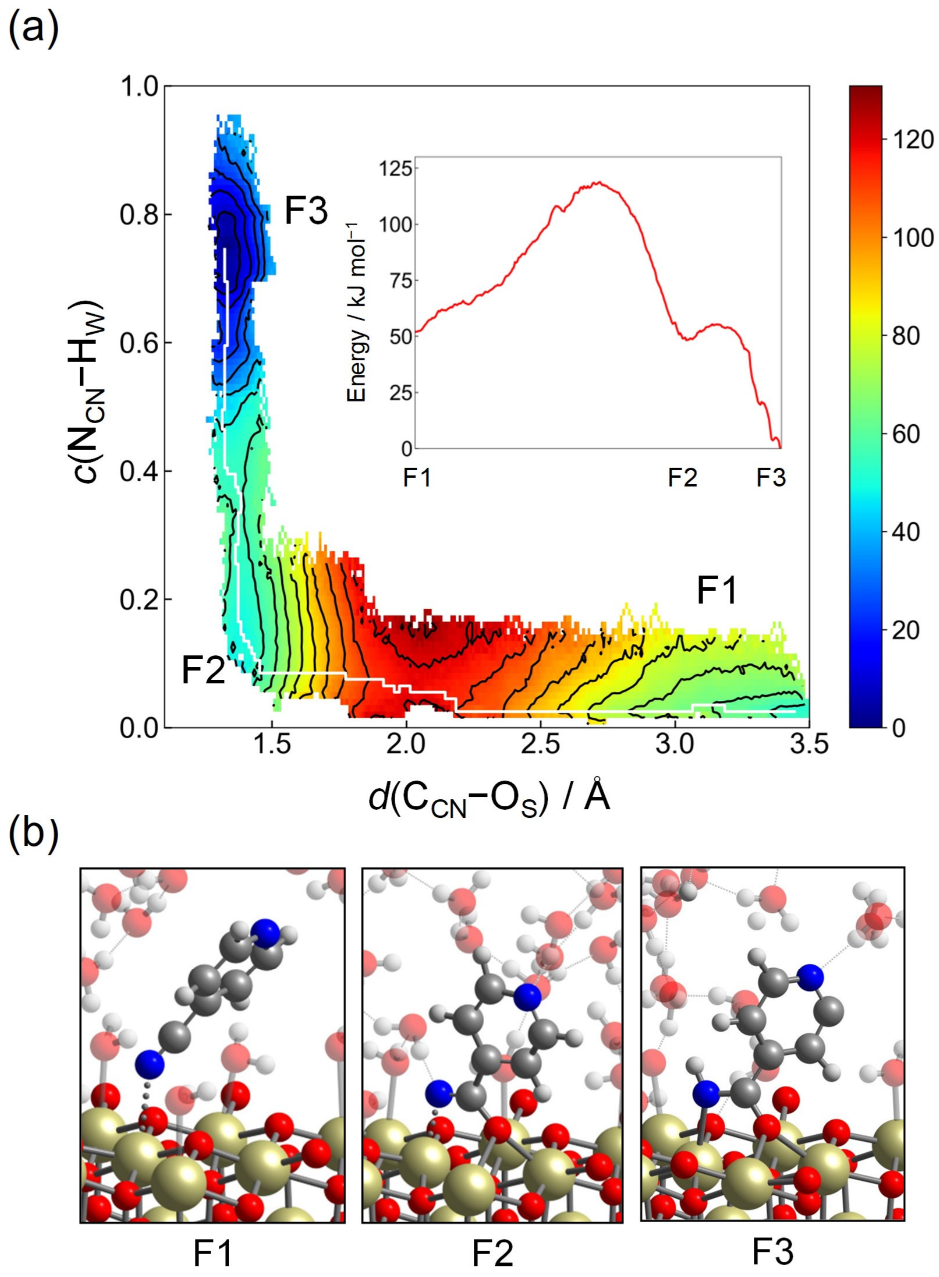

Figure S2. (a) Contour plot of the free energy surface (given in kJ/mol) for adsorption process of 4-cyanopyridine from the molecular adsorption state. The minimum free energy pathway, which is determined by the MULE algorithm, is shown in grey. The inset shows the free energy profile along the minimum free energy pathway. (b) Snapshots of representative configuration near **F1**, **F2**, and **F3**.

Hydration of 2-Cyanopyridine in the Bulk Water

The simulation cell of $a = b = c = 12.0$ Å in the cubic box was employed. The 50 water molecules were placed in the simulation cell to reproduce the density of bulk water. Two CVs, $d(\mathrm{C_{CN}}{-}\mathrm{O_W})$ and $c(\mathrm{O_W}{-}\mathrm{H_W})$, are employed to obtain the free energy profiles, and the force constants of umbrella potential were set to be the same values as in **Step 2-(I)** and **Step 2-(III)**. The contour plot of the free energy surface spanned by $d(\mathrm{C_{CN}}{-}\mathrm{O_W})$ and $c(\mathrm{O_W}{-}\mathrm{H_W})$ is shown in Figure S3(a). The reactant corresponds to **E1**, and the transition state region is located at $d(\mathrm{C_{CN}}{-}\mathrm{O_W}) \approx 1.7$ Å and $c(\mathrm{O_W}{-}\mathrm{H_W}) \approx 1.2$ (see for snapshot given in Figure S3(b)), where the reaction involves a concomitant proton transfer of an attacking water molecule to the neighboring water molecules, and the free energy barrier is estimated as 120 kJ/mol.

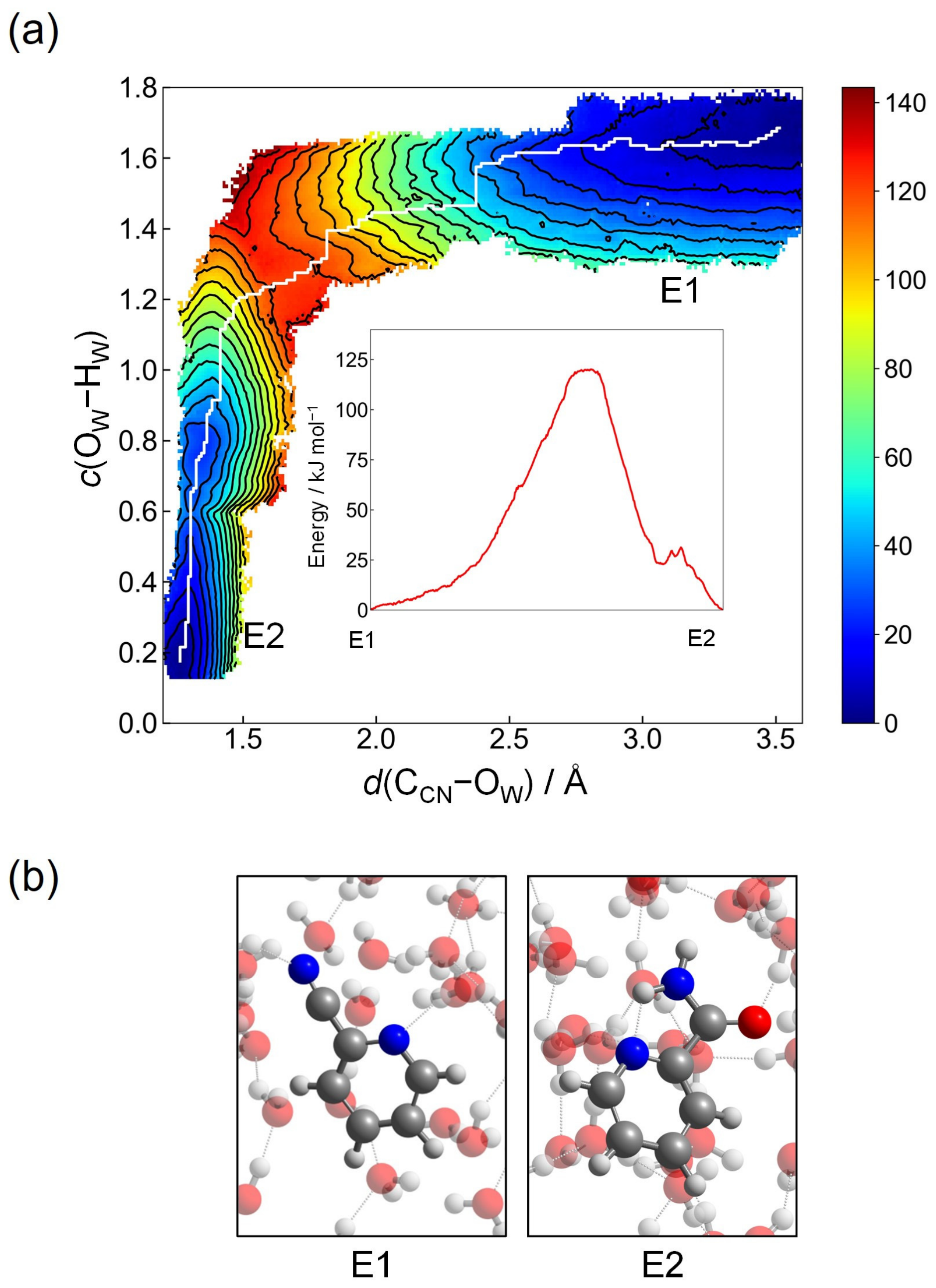

Figure S3. Contour plot of the free energy surface (given in kJ/mol) for the hydration reaction of 2-cyanopyridine in bulk water. The minimum free energy pathway, which is determined by the MULE algorithm, is shown in white. The inset shows the free energy profile along the minimum free energy pathway. (b) Snapshots of representative configuration near **E1** and **E2**.

Comparison between the potential (DFT) energy and free energy calculations.

For the adsorption process of **Step-1-(II)**, energy differences associated with **A1** → **A2** are compared. The potential (DFT) energy difference is calculated to be −21 kJ/mol (see Figure 2), while the free energy difference is given as −28 kJ/mol (see the inset of Figure 4(a)), which is relatively close to each other. It is suggested that the effects of the solvents and thermodynamic are not particularly significant. A comparison of the **A2** → **A3** step is not feasible as it involves a proton hopping from surrounding water molecules to 2-cyanopyridine, which is not amenable to static calculations. Additionally, **B1−II** → **B2** in **Step-2-(I)** is considered. The energy difference is found to be −40 kJ/mol and +5 kJ/mol for static calculations and free energy calculations, respectively, where a significant discrepancy is observed. The influence of surrounding molecules (hydrogen bond) is expected to be substantial for this step.

Experimental Details

Materials

Preparation of pure CeO_2 catalyst was carried out by calcining cerium oxide HS (Daiichi Kigenso, Japan) for 3 h in air at 873 K. The specific surface area (BET method) of the CeO_2 was 90 m^2 g^{-1}, and a crystalline size of 9.7 nm was estimated by XRD (Figure S8). The catalyst has (111) faces, which were confirmed by TEM analyses (Figure S9). The purity of the CeO_2 is 99.97%. All the chemicals for organic reactions were commercially available and were used without further purification. 2-Cyanopyridine (Tokyo Chemical Industry Co., Ltd.), 1,4-dioxane (FUJIFILM Wako Chemicals Corporation), $H_2{}^{18}O$ (TAIYO NIPPON SANSO Corporation).

Catalyst characterizations

X-ray diffraction (XRD) patterns were recorded by a diffractometer (MiniFlex600, Rigaku). Cu Kα (λ = 0.154 nm, 45 kV, 40 mA) radiation was used as an X-ray source. XRD measurements of the samples were conducted under air. Specific surface areas of metal oxides were measured with BELSORP MINI X (MicrotracBEL Corporation) by using BET method. A field-emission scanning transmission electron microscope (FE-STEM) image was taken using JEM-ARM200F. The samples were dispersed in ethanol and placed on Cu grids under air.

Preparation of ^{18}O-substituted CeO_2

CeO_2 was set on the glass tube in a powder form and reduced at 873 K under H_2 flow (30 mL/min) for 10 min. After that, H_2 flow was changed to N_2 flow (30 mL/min), and $^{18}O_2$ (100 ml) was introduced to the reduced CeO_2 at 873 K. The CeO_2 was cooled to room temperature under N_2 flow (30 mL/min), and N_2 flow was stopped. N_2 gas in the glass tube was substituted by $^{18}O_2$ (50 mL), and the sample was exposed to $^{18}O_2$ for 10 min, providing ^{18}O-substituted CeO_2.

Hydration of 2-cyanopyridine over CeO_2 or ^{18}O-substituted CeO_2

Hydration of 2-cyanopyridine was conducted as follows: CeO_2 or ^{18}O-substituted CeO_2 (100 mg, 0.58 mmol) was added to the mixture of 2-cyanopyridine (104 mg, 1.0 mmol) in H_2O (5.0 g) in a reaction vessel equipped with a condenser under air. The resulting mixture was vigorously stirred at appropriate temperatures (279 and 274 K). The conversion and yield of the hydrated products were determined based on 2-cyanopyridine and picolinamide by GC using 1,4-dioxane as an internal standard. The products were identified by GC-MS (Shimazu QP2020) and compared with commercially pure products.

Hydration of 2-cyanopyridine in H_2O or $H_2{}^{18}O$

Hydration of 2-cyanopyridine was conducted as follows: CeO_2 (100 mg, 0.58 mmol) was added to the mixture of 2-cyanopyridine (104 mg, 1.0 mmol) in H_2O or $H_2{}^{18}O$ (3.0 g) in a reaction vessel equipped with a condenser under air. The resulting mixture was vigorously stirred at appropriate temperatures (278 K). The conversion and yield of the hydrated products were determined based on 2-

cyanopyridine and picolinamide by GC (Shimadzu GC-2014) with a CP-Sil 5 CB capillary column (Agilent J&W) using 1,4-dioxane as an internal standard. The products were identified by GC-MS (Shimazu QP2020) and compared with commercially pure products.

O_2 adsorption

The amount of oxygen displaced by the reduction and subsequent oxidation treatment of CeO_2 was estimated by the O_2 uptake using pulse-adsorption of O_2 with Belcat II (MicrotracBEL, Japan) with a thermal conductivity detector (TCD) and Q-Mass (BELMASS, MicrotracBEL, Japan). The sample weight was 200 mg. Before the measurements, the CeO_2 was reduced at 873 K under H_2 flow (30 mL min^{-1}) for 10 min. After cooling to room temperature under He flow (30 mL min^{-1}), a series of O_2 pulses (77 mm^3) are injected at 313 K with an interval of 2 min until the amount of exit O_2 pulses reaches a steady state value.

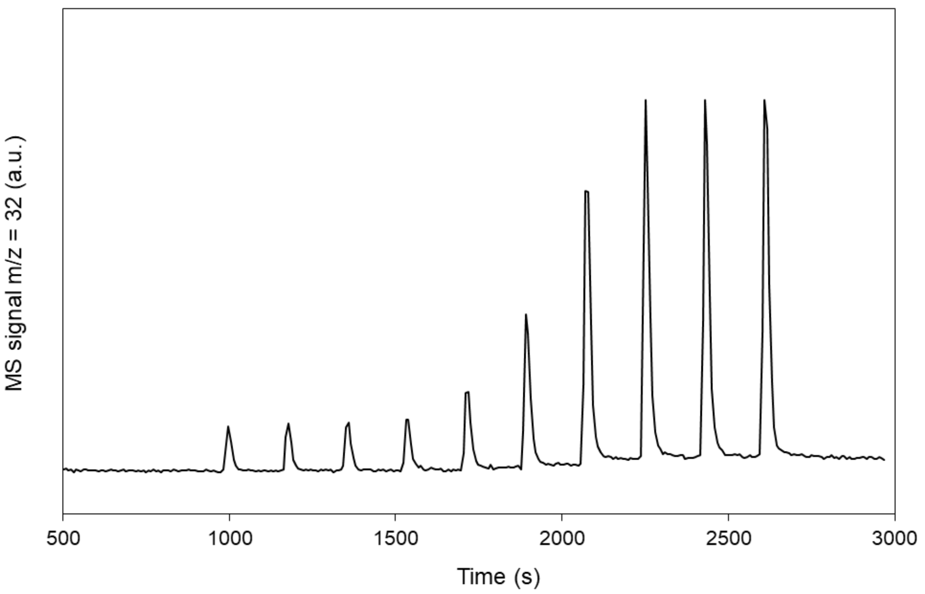

Figure S4. MS spectrum of O_2 adsorption of the reduced CeO_2. Reduction conditions: 873 K, H_2 (30 mL min^{-1}), 10 min. Measurement conditions: O_2 pulse size 77 mm^3, 313 K.

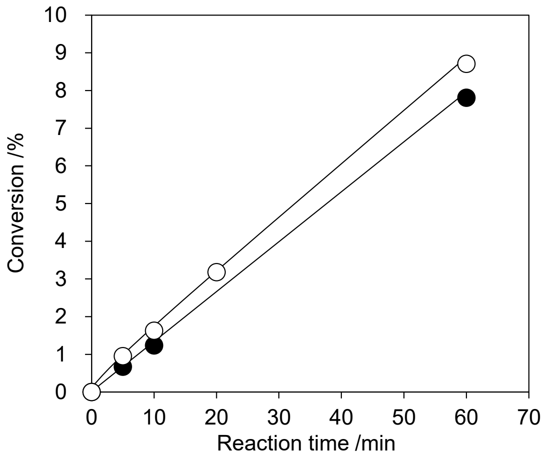

Figure S5. Conversion of hydration of 2-cyanopyridine in $H_2{}^{18}O$ and H_2O over CeO_2 at 278 K. Black circle: H_2O, white circle: $H_2{}^{18}O$. Reaction conditions: CeO_2 100 mg (0.58 mmol), 2-cyanopyridine 104 mg (1.0 mmol), H_2O or $H_2{}^{18}O$ 3 g, 278 K.

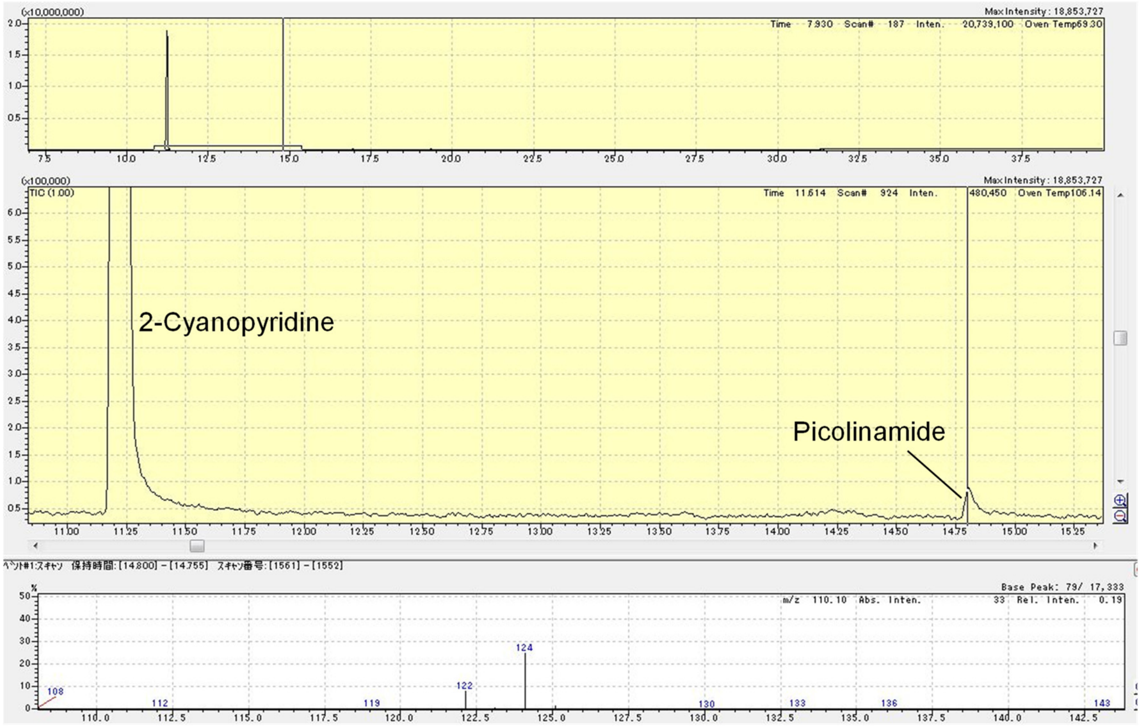

Figure S6. Typical example of the mass spectrum of the produced picolinamide in hydration of 2-cyanopyridine in $H_2{}^{18}O$ over CeO_2 (5 min). Reaction conditions: CeO_2 100 mg (0.58 mmol), 2-cyanopyridine 104 mg (1.0 mmol), $H_2{}^{18}O$ 3 g, 278 K.

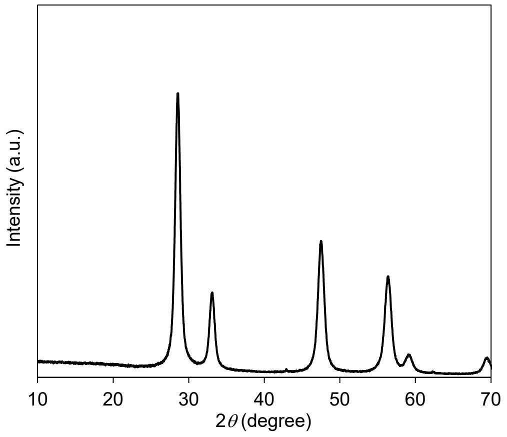

Figure S7. XRD analyses of CeO_2 calcined at 873 K and air.

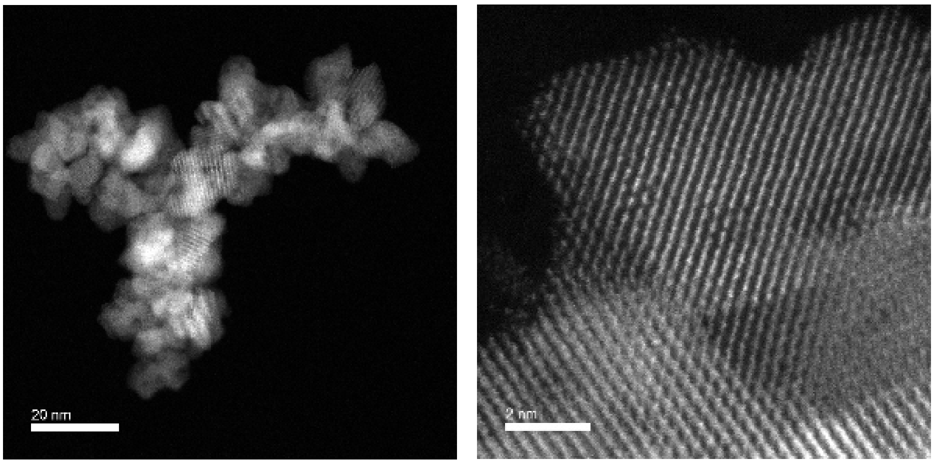

Figure S8. STEM images of CeO_2 calcined at 873 K

Table S1. Detailed results of hydration of 2-cyanopyridine with CeO_2 and ^{18}O-substituted CeO_2 catalysts at 279 K and 274 K.

Entry	*T* /K	Catalyst	*t* /s	Conv. %	Sel. /%	Intensity[a] 122	Intensity[a] 124	The ratio of peak intensities (124/122) /%	$^{18}O/^{16}O$ ratio in picolinamide*
1	279	CeO_2	0	0	–	–	–	–	–
2			120	8.5	>99.9	80801	327	0.40	0
3			300	17.6	>99.9	192372	729	0.38	0
4			600	30.6	>99.9	140279	548	0.39	0
5	279	^{18}O-substituted CeO_2	0	0	–	–	–	–	–
6			60	6.2	>99.9	19396	353	1.82	1.41
7			180	12.6	>99.9	49807	639	1.28	0.88
8			300	18.7	>99.9	49830	569	1.14	0.74
9			600	31.2	>99.9	207919	1340	0.64	0.24
10	274	CeO_2	0	0	–	–	–	–	–
11			120	6.0	>99.9	94245	410	0.44	0
12			600	22.3	>99.9	60226	282	0.47	0
13	274	^{18}O-substituted CeO_2	0	0	–	–	–	–	–
14			20	1.5	>99.9	2636	49	1.86	1.45
15			60	2.8	>99.9	4832	97	2.01	1.60
16			120	4.9	>99.9	19396	353	1.82	1.41
17			300	10.6	>99.9	49807	639	1.28	0.88

Reaction conditions (b): CeO_2 or ^{18}O-substituted CeO_2 100 mg (0.58 mmol), 2-cyanopyridine 104 mg (1.0 mmol), H_2O 5 g, 279 or 274 K.

*The $^{18}O/^{16}O$ ratio in picolinamide is calculated by subtracting the influence of natural origin based on the reference reaction with CeO_2 (The ratio of peak intensities (124/122) is ~0.4, entries 1−4 and 10−13).

Table S2. Detailed data of **Figure 10**

Conv.[a)] /%	^{18}O amount in picolinamide derived from ^{18}O-substituted CeO_2[b)] /mmol	^{16}O amount in picolinamide[c)] /mmol	$^{18}O/^{16}O$ in picolinamide
1.5	0.21	14.7	1.45
2.8	0.44	27.4	1.60
4.9	0.68	48.1	1.41
6.2	0.86	60.9	1.41
10.6	0.92	105	0.88
12.6	1.09	124	0.88
18.7	1.36	185	0.74
31.2	0.74	310	0.24

[a)] Results are from **Figure 9** and **Table S1**.

[b)] ^{18}O amount in picolinamide derived from ^{18}O-substituted CeO_2 is calculated by the following equation: (^{18}O amount in picolinamide derived from ^{18}O-substituted CeO_2 (mmol)) = (^{18}O amount in picolinamide (mmol)) – (Amount derived from the natural abundance of the isotopes (mmol)). ^{18}O amount in picolinamide is calculated from the mass intensities of 122 and 124 of the products (**Table S1**).

[c)] ^{16}O amount in picolinamide is calculated by the following equation: (^{16}O amount in picolinamide (mmol)) = (Total produced picolinamide (mmol)) – (^{18}O amount in picolinamide (mmol)).

Table S3. Detailed results of hydration of 2-cyanopyridine in $H_2{}^{18}O$ and H_2O over CeO_2 at 278 K.

Solvent	*t* /min	Conversion %	Selectivity /%	Intensity[a]		The peak intensity ratio (122/124) /%
				122	124	
$H_2{}^{18}O$	0	0.0	–	–	–	–
	5	0.95	>99.9	1425	4237	33.6
	10	1.6	>99.9	728	4810	15.1
	20	3.2	>99.9	696	5951	11.7
	60	8.7	>99.9	674	8398	8.0
H_2O	0	0	–	–	–	–
	5	0.67	>99.9	–	–	–
	10	1.23	>99.9	–	–	–
	60	7.8	>99.9	–	–	–

Reaction conditions: CeO_2 100 mg (0.58 mmol), 2-cyanopyridine 104 mg (1.0 mmol), H_2O or $H_2{}^{18}O$ 3 g, 278 K.

[a] Intensity was determined by GC-MS analyses.

References

(1) Tamura, M.; Kishi, R.; Nakayama, A.; Nakagawa, Y.; Hasegawa, J.; Tomishige, K. Formation of a New, Strongly Basic Nitrogen Anion by Metal Oxide Modification. *J. Am. Chem. Soc.* **2017**, *139*, 11857–11867.
